# Bioprinting of hepatic tissue model using photocrosslinkable dECM-containing composite hydrogel

**DOI:** 10.1016/j.mtbio.2025.101824

**Published:** 2025-05-03

**Authors:** Nima Tabatabaei Rezaei, Hitendra Kumar, Hongqun Liu, Ashna Rajeev, Giovanniantonio Natale, Samuel S. Lee, Simon S. Park, Keekyoung Kim

**Affiliations:** a Department of Mechanical and Manufacturing Engineering, University of Calgary, Calgary, Alberta, T2N 1N4, Canada; b Department of Biosciences and Biomedical Engineering, Indian Institute of Technology Indore, Indore, Madhya Pradesh, 453552, India; c Liver Unit, Cumming School of Medicine, University of Calgary, Calgary, Alberta, T2N 1N4, Canada; d Department of Chemical Engineering, University of Waterloo, Waterloo, Ontario, N2L 3G1, Canada; e Department of Chemical & Petroleum Engineering, Schulich School of Engineering, University of Calgary, Calgary, Alberta, T2N 1N4, Canada; f Department of Biomedical Engineering, University of Calgary, Calgary, Alberta, T2N 1N4, Canada

**Keywords:** Liver decellularized extracellular matrix, Bioprinting, Visible light photo crosslinking, Liver functionality, Drug screening

## Abstract

The liver, as one of the vital organs in the body, plays a crucial role in various bodily functions. Numerous factors can cause liver damage, that the sole remedy for severe liver conditions is transplantation of healthy liver tissue. In response to the transplantation challenges, innovative approaches involving hydrogel-based technologies have emerged, leading to the creation of highly functionalized tissues. The development of three-dimensional printing and patterning of cell-laden biomaterial matrices offers promising advances for creating tissue-specific structures in tissue engineering and bioprinting. However, the matrix materials currently used in bioprinting liver microtissue often fail to capture the complexity of the natural extracellular matrix (ECM), hindering their ability to restore innate cellular shapes and functions. Liver ECM-based hydrogels are increasingly recognized for their potential as biomimetic three-dimensional (3D) cell culture systems that facilitate the exploration of liver disease, metabolism, and toxicity mechanisms. Yet, the conventional production of these hydrogels relies on slow thermal gelation processes, which restrict the manipulation of their mechanical characteristics. In this research, we introduce a novel approach with a functionalized photocrosslinkable liver decellularized extracellular matrix (dECM). By combining liver dECM methacrylate (LdMA) with gelatin methacrylate (GelMA), we achieved accelerated crosslinking under visible light irradiation and the ability to tune the mechanical, rheological, and physiological properties of the material. We encapsulated human hepatocellular carcinoma cells within an optimal concentration of the GelMA-LdMA hybrid hydrogel and examined cell proliferation and function over an extended period. The results demonstrated that the GelMA-LdMA hybrid hydrogel effectively sustains cell viability over an extended period while promoting enhanced liver cell proliferation, suggesting its potential for drug screening applications and liver cancer metastasis research. Notably, albumin secretion in the dECM-based hydrogel was approximately 40 % higher compared to the control GelMA sample. Furthermore, when evaluating acetaminophen-induced hepatotoxicity, the hybrid hydrogel showed a promising drug response, with significant upregulation of the drug metabolism-related gene cytochrome P450-1A2 (CYP1A2). Overall, the dECM-based hepatic tissue model demonstrated excellent biofunctionality and responsiveness to drug treatment, making it a promising candidate for in vitro toxicological studies.

## Graphical abstract

Created in BioRender. Kim, K. (2025) https://BioRender.com/j87l183.

**Figure:**
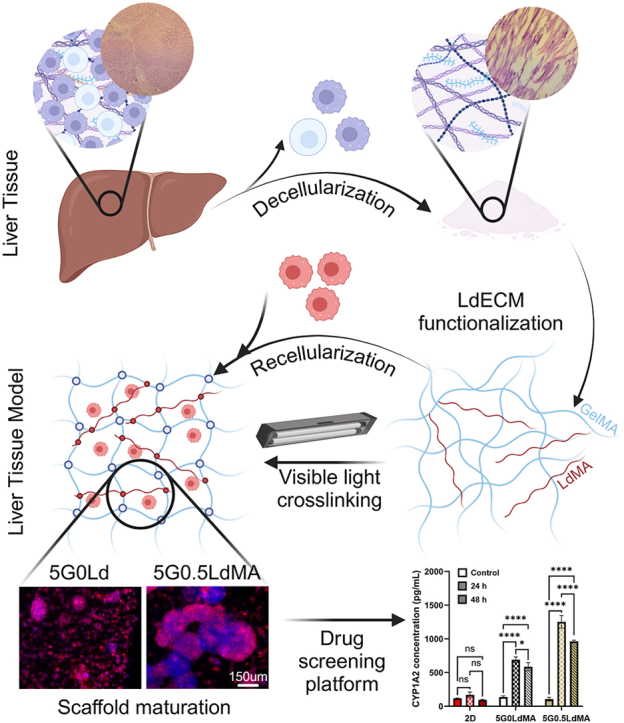

**Table:** Abbreviations

3D	three-dimensional
GelMA	gelatin methacrylate
ECM	Extracellular matrix
dECM	decellularized extracellular matrix
LdMA	Liver decellularized extracellular matrix methacrylate
SDS	sodium dodecyl sulfate
DLP	digital light processing
EY	eosin Y
TEOA	triethanolamine
SEM	scanning electron microscopy
PBS	phosphate buffered saline
H&E	hematoxylin and eosin
GMA	glycidyl methacrylate

## 1 Introduction

The liver is an essential organ responsible for maintaining the body's biochemical balance, including the synthesis of blood proteins, glucose metabolism, and the detoxification of various substances [[1], [2], [3]]. Despite its critical functions, the incidence of liver disease-related fatalities is on the rise globally [4]. Liver transplantation or cell therapy techniques such as hepatocyte transplantation [5] has shown promising potential to be used as viable treatment for end-stage liver disease that is limited by the scarcity of donor organs and challenges with cell engraftment [6]. Consequently, addressing end-stage liver disease remains a significant hurdle, filled with uncertainties regarding effective treatment methods. Advancements in bioengineering, particularly through tissue engineering techniques, have led to the exploration of new solutions. These include the creation of transplantable liver microtissues or entirely bioengineered livers, offering promising alternatives for the long-term restoration of liver function [2,7].

As the newly emerged class of biomaterials, tissue engineering approaches have effectively harnessed the decellularized extracellular matrix (dECM) to mimic the native cellular microenvironment for tissue fabrication in vitro [8,9]. The ECM's unique three-dimensional (3D) microstructure and organization are pivotal in regulating cell behavior, communication, and maintaining tissue integrity and functionality [10]. A prevalent method for obtaining dECM employs various chemical agents, including ionic and non-ionic detergents, acids and bases, alcohols, and chelating agents [11]. By integrating these tissue-specific extracellular matrix (ECM) components into 3D model systems, it is possible to facilitate the development of tissues that more accurately resemble their physiological counterparts in terms of function and structure [12]. Lee et al. [7] developed a liver dECM-based bioink and bioprinted a liver tissue model using extrusion bioprinting, encapsulating HepG2 cells. Their study demonstrated that cells proliferated more rapidly in dECM hydrogels compared to collagen (control group), with higher albumin and urea secretion over a 7-day culture period. Moreover, in several studies, dECM has been combined with thermally crosslinkable hydrogels to enhance control over the system's mechanical properties. Kim et al. [13] incorporated liver dECM into gelatin to assess printability. However, when mouse primary hepatocytes were encapsulated in their bioink, the secretion of liver-specific markers progressively declined over two weeks of culture, indicating a lack of long-term culture stability in their system. These studies rely solely on thermal crosslinking of dECM limiting the ability to achieve uniform constructs with tunable mechanical properties [14,15]. To address this, various studies have incorporated additional polymer matrices to blend with dECM. For instance, You et al. [16] blended liver dECM with gelatin and alginate, utilizing ionic crosslinking with CaCl_2_ to regulate crosslinking kinetics and scaffold properties, which enabled the encapsulation of rat bone mesenchymal stem cells.

The structure of the fabricated scaffolds, along with the composition of the scaffold biomaterial, plays an equally important role in defining the microenvironment and, consequently, the function of the fabricated tissues. While progress has been made in liver tissue engineering using extrusion-based bioprinting techniques, these efforts are hindered by challenges such as low printing resolution and compromised structural integrity [17]. These issues stem from the inherent limitations of extrusion-based printing methods and the inadequate mechanical properties of dECM-based bioinks [18]. To address these limitations, photocuring-based biofabrication methods, including digital light processing (DLP) [19,20] have been employed to create liver models for various biomedical applications. DLP printing can achieve high-resolution hydrogel structures with intricate architectures and can enhance the mechanical properties of the printed hybrid and composite hydrogels [20]. Mao et al. [21] blended liver dECM with GelMA and used lithium phenyl-2,4,6-trimethylbenzoylphosphinate (LAP) as a photoinitiator to develop a photocrosslinkable bioink suitable for DLP bioprinting. Their study investigated the impact of dECM incorporation into GelMA on the mechanical and physical properties of the resulting scaffold, as well as its effects on encapsulated hepatocytes. Their findings demonstrated that cell proliferation was enhanced in the groups containing liver dECM, highlighting its potential to improve hepatocyte viability and function within 3D bioprinted liver models. However, DLP printing involves the use of potentially harmful photoinitiators (e.g. lithium phenyl-2,4,6-trimethylbenzoylphosphinate, LAP) that, along with UV light, can unpredictably affect cell behavior. As a result, recently, there has been a growing interest in exploring photoinitiators that activate under visible light excitation [22]. Eosin Y (EY) offers several advantages over LAP as a photoinitiator, particularly for biofabrication applications. EY enables cell-friendly polymerization under milder conditions (e.g., green light), whereas LAP requires higher-energy UV or blue light, which may negatively impact cell viability. Additionally, EY has higher water solubility and is effective at lower concentrations, reducing cytotoxicity while maintaining efficient crosslinking. These properties make EY a promising alternative for biocompatible hydrogel-based tissue engineering applications [23,24].

In order to swiftly produce hydrogels that exhibit controlled gelation processes, the technique of functionalizing natural polymers with photocurable components has been utilized [[25], [26], [27]]. This method combines the inherent benefits of natural polymers—such as biocompatibility and biodegradability—with the consistent physicochemical properties provided through chemical functionalization. The resulting hydrogels are capable of forming controllable, reproducible, and biologically relevant three-dimensional tissue models. However, existing ECM-based hydrogels for liver tissue have limited ability to manipulate their physical characteristics, and there have been relatively few attempts to enhance the physicochemical properties of liver dECM hydrogels [21,28].

Several studies have blended dECM with GelMA as reviewed previously, but due to the lack of proper functional groups, dECM remains physically entangled in the GelMA network without forming covalent bonds (leading to an interpenetrating polymer network (IPN)), resulting in rapid degradation and loss of mechanical integrity over time. Addressing the critical need for an enhanced liver matrix-derived bioink that guarantees superior printability, cell viability, and functionality, this study adopts a chemically modified liver dECM to be used for tissue engineering. In this work, the solublized decellularized liver tissues were then methacrylated, resulting in the formation of liver dECM methacrylate (LdMA). This was further mixed with gelatin methacrylate (GelMA) to create hybrid hydrogels upon photocrosslinking. In contrast to liver dECM, LdMA contains methacrylate groups, allowing it to covalently crosslink with GelMA during photocrosslinking, anticipating to significantly improve the mechanical strength, stability, and degradation control. By integrating LdMA with GelMA, we were able to bioprint liver tissue models with adjustable and enhanced physical, mechanical and biological attributes. The Ld-based hydrogels underwent comprehensive characterization to assess their functionalization degree, physical characteristics, and cytocompatibility. Ultimately, by encapsulating liver cells within the developed scaffold and monitoring their functionality over extended culture periods, we demonstrated the scaffold's efficacy towards liver tissue engineering applications. Additionally, considering the crucial function of liver metabolism in preclinical toxicity testing for the development of new drugs and therapeutic agents [29,30], we examined whether our 3D liver platform could serve as a more sensitive and effective tool, compared to traditional 2D cultures, for more realistic screening of hepatotoxic compounds.

## 2 Materials and methods

### 2.1 Porcine liver decellularization

Fresh porcine liver tissues were purchased from a local butcher shop and transported on ice to the lab and stored in −80 °C freezer prior to decellularization process. Previously reported protocols were modified to prepare the liver dECM [[31], [32], [33]]. In brief, frozen liver tissues were thawed and cut into small cubic pieces (less than 1 mm) with scissors and washed in distilled water for 3 h to remove blood. Next, the samples were soaked in 1 % (w/v) Triton X-100 (VWR, Mississauga, ON, Canada) for 24 h, followed by treatment with 0.1 % (wt/vol) sodium dodecyl sulfate (SDS) (VWR, Mississauga, ON, Canada) solution for 48 h, replacing fresh solution after first 24 h. A solution to liver tissue ratio of 10:1 was maintained throughout all the washing steps. Then, tissue pieces were rinsed thoroughly with distilled water several times and blended with high speed to have uniform product. Finally, blended liver tissue was separated by centrifuging at 3500 rpm for 3 min and was stored at −80 °C and then lyophilized to obtain final dECM.

### 2.2 Decellularization assessment

To visualize and investigate residual cells and microarchitecture, both native and decellularized porcine liver tissues were fixed in 4 % (w/v) paraformaldehyde solution at room temperature for 24 h followed by sequential immersing in 100 %, 95 %, 90 %, 75 %, 70 % and 50 % (v/v) ethanol overnight, and then embedded in paraffin. Paraffined sections were cut into thin slices of 5–7 μm thickness for further staining steps. Then, tissue sections were stained by hematoxylin and eosin (H&E) (VWR, Mississauga, ON, Canada) and Masson's trichrome staining (Polysciences Inc., Warrington, PA, USA) in order to observe cell component residues and collagen distribution, respectively. The H&E and Masson trichrome staining samples were examined using an inverted microscope (ECHO revolve).

Slices from the liver specimens and the dECM samples were subjected to DAPI staining. This technique was employed to visualize the nuclei within the liver tissues, and to assess the presence or absence of nuclei in the dECM samples, respectively. Fluorescence microscopy images were acquired using an inverted fluorescence microscope (ECHO revolve) using the DAPI fluorescence channel ($\lambda_{ex}350nm/\lambda_{em}465nm$).

DNA and total protein concentration (TPC) quantified content were measured using the PicoGreen dsDNA Assay Kit (ThermoFisher, Waltham, Massachusetts, USA) and TPC kit (ThermoFisher, Waltham, Massachusetts, USA), respectively, to indicate residual cells and protein content in the dECM (n = 3). Briefly, 50 mg of native liver and lyophilized liver dECM were added to 1 mL of pepsin digest solution (Ward's Science, ON, Canada) with a concentration of 0.1 mg/mL and homogenized with homogenizer (FSH-2A High Speed Homogenizer) at 20000 rpm. Then samples were vortexed and kept in 65 °C water bath for 6 h followed by centrifuging at 4000 rpm for 15 min to collect the supernatant. A microplate reader (SpectraMax ® iD3 Microplate Readers) was used for fluorescence intensity measurement 480 nm excitation and absorbance measurement under 560 nm for DNA and TPC kits, respectively.

### 2.3 dECM functionalization

Lyophilized dECM was digested in 0.5 M of acetic acid (MilliporeSigma, Oakville, ON, Canada) and 10 mg pepsin solution per 100 mg dECM for 3 days at room temperature to obtain uniform solution. After complete digestion, using 1 M NaOH (MilliporeSigma, Oakville, ON, Canada) solution, the dECM solution's pH was adjusted approximately 8.5. Then, 300 μL of glycidyl methacrylate (GMA, MilliporeSigma, Oakville, ON, Canada) per 0.5 g dECM in the solution was added dropwise at room temperature and agitated aggressively for 24 h. The resulting LdMA was dialyzed in a 12–14 kDa dialysis tubing (ThermoFisher, Waltham, Massachusetts, USA) against distilled water for 3 days with water changed twice a day. Then, the solution was lyophilized at −84 °C for another 3 days. The lyophilized LdMA were stored at −80 °C for further use.

The degree of substitution of the synthesized LdMA was measured using proton nuclear magnetic resonance (1H NMR) as reported previously [34,35]. LdMA solution was prepared with a concentration of 0.5 % (w/v) 1 mL of deuterium oxide (ThermoFisher, Waltham, Massachusetts, USA). Next, a 600 MHz NMR spectrometer (Bruker 600 MHz Avance III Spectrometer) was used to record the 1H NMR spectra for the synthesized LdMA samples. The internal reference was set to glycine α-CH protons signal (3.5–3.8 ppm). The methacryloyl proton peaks located at 5.77 ppm, 5.42 ppm, and 1.94 ppm were integrated to compute the degree of substitution [36].

### 2.4 Preparation of prepolymer solution and photopatterning system

GelMA was synthesized following a previously reported protocol [37]. In brief, 5 g of powdered gelatin from porcine skin (Type A, Bloom strength 300, Sigma-Aldrich, St. Louis, MO, USA) was dissolved in 50 mL of RO purified water. After complete dissolution at 48–50 ᵒC, 9–10 mL of GMA (MilliporeSigma, Oakville, ON, Canada) was added to the gelatin solution dropwise. The solution was then maintained at 48–50 °C with constant stirring at 750 rpm for 12 h. Upon completion of the reaction, the solution was transferred into dialysis tubing (12–14 kDa) to be dialyzed against distilled water for 3 days with water changed twice a day. After 3 days, the remaining solution was frozen and then lyophilized to obtain a foamy solid of GelMA. The lyophilized samples were stored at −20 °C for further use. In order to prepare hybrid prepolymer solution, 5 % (w/v) GelMA and different concentrations of Ld or LdMA were dissolved in PBS, as listed in Table 1. For this purpose, first 5 % (w/v) GelMA solution was prepared and then different amounts of Ld or LdMA were added to the solution and stirred overnight at room temperature to achieve a homogeneous prepolymer solution. Next, the photoinitiators for visible light crosslinking were added to the prepolymer solutions. An eosin Y (EY)-based two-component photoinitiator as reported in earlier studies was used for the crosslinking of prepared bioinks [37,38]. The two-component photoinitiator was made up of 0.02 mM EY disodium salt (VWR, Mississauga, ON, Canada) and 0.2 % w/v triethanolamine (TEOA) (MilliporeSigma, Oakville, ON, Canada). After preparing GelMA-LdMA solution, EY and TEOA were added to reach the final concentration of 1 % (v/v) for each component. The designed structures were formed by exposing the prepolymer solutions to visible light (wavelength: 400–700 nm, intensity: 80.8 mW cm^−2^) in the modified custom-made DLP bioprinting system reported earlier [38,39] as shown in Figure S10A. The exposure duration was varied for each bioink combination based on their photocrosslinking characteristics. Circular vats with 500 μm depth were used to crosslink the bioinks and form the designed structures in one step.

**Table 1:** Different compositions of dECM-based bioinks.

Ink code	GelMA concentration	Ld concentration	LdMA concentration
**5G**	5 %	0 %	0 %
**5G0.5LdMA**	5 %	0 %	0.5 %
**5G1LdMA**	5 %	0 %	1 %
**5G2LdMA**	5 %	0 %	2 %
**5G0.5Ld**	5 %	0.5 %	0 %
**5G1Ld**	5 %	1 %	0 %
**5G2Ld**	5 %	2 %	0 %

### 2.5 Measurements of mechanical, swelling and degradation properties

The mechanical properties of the hydrogels were assessed by evaluating the compressive modulus of the crosslinked hydrogels as a measure of mechanical stiffness. The probe used for the compression was a flat-ended rigid cylinder with 12.5 mm diameter. In order to prepare samples, a 2 mL solution of the hydrogel precursor with different concentration of LdMA was prepared and transferred into cylindrical molds with 8 mm diameter and 8 mm depth. The prepolymer solution was added in each well. The prepolymer solution was then crosslinked in the DLP bioprinting system for 5 min due to the large volume using the DLP projector to ensure the whole volume was crosslinked. Additionally, to observe the influence of functionalization of dECM on the mechanical properties of the hybrid hydrogels, unmodified Ld was also used to prepare prepolymer solutions with the same concentration and crosslinked under same conditions as GelMA-LdMA prepolymer solutions. Afterwards, a vertical axis micromechanical testing machine along with the custom flat-cylindrical probe was used to record the force vs. displacement plots for hydrogels under compression. Up to 70 % compression was applied on the hydrogel surface to obtain the force-displacement plot. Using initial dimensions of the hydrogel samples and a custom-made MATLAB script, compression modulus was determined using the slope of the linear region of initial 10 % of strain.

The swelling ratio of the crosslinked hydrogel samples was measured to evaluate the water uptake capability. To describe the procedure briefly, 250 μL of different concentrations of hydrogel solutions were casted in 8 mm diameter and 8 mm deep cylindrical mold and crosslinked under visible light using the DLP system for 5 min. Subsequently, the crosslinked hydrogels were immersed in PBS and kept in an incubator at 37 °C and 5 % CO_2_for 24 h. Each sample's hydrated weight (w_w_) was recorded at hydration equilibrium. Afterwards, they were frozen in a −80 °C freezer and lyophilized for 48 h before their dry weight (w_d_) was measured. Finally, the following equation was used to calculate the swelling ratio (n = 5):

$$Swelling\;ratio=\frac{w_{w}-w_{d}}{w_{d}}\times 100$$

In order to investigate the hybrid hydrogels' degradation behaviour, 750 μl of GelMA-LdMA hydrogels were cast in 96-well plates and cured for 5 min under visible light. In addition, a sample containing 5 % GelMA and 0.5 % unmodified and solublized Ld was used to demonstrate the influence of methacrylation on the degradation properties of the hydrogels. All the samples were lyophilized for 48 h and weighed to get the initial weight (w_0_), and then were immersed in PBS at 37 °C for 24 h. The swollen samples were placed in a 48-well plate with collagenase-I enzyme solution (50 U/mL) and incubated at 37 °C for hydrogel degradation study. Three samples were collected every 1 h, frozen at −80^o^C and lyophilized for 24 h. The lyophilized samples were weighed to obtain the remaining hydrogel matrix weight (w_r_) after degradation. The biodegradation property (Q_d_) of hydrogels was indicated by the percentage of the remaining weight of the disks after degradation as:

$$Q_{d}=\frac{w_{r}}{w_{0}}\times 100$$

### 2.6 Scanning electron microscopy (SEM) and porosity quantification

Scanning electron microscopy (SEM) was used to investigate the microscale structure of the GelMA-LdMA hybrid hydrogels, . For this characterization, 250 μL prepolymer solution was prepared for each hydrogel group at defined concentrations and, crosslinked with visible light irradiation. Subsequently, the samples were frozen in a −80 °C freezer followed by lyophilization for two days. The lyophilized samples were then sputter-coated with carbon sputtering machine and imaged using SEM (Phenom Pro X).

### 2.7 Crosslinking kinetic evaluation and rheological characterization

The rheology characteristics of GelMA and different types LdMA-based hybrid bioinks were evaluated using a rheometer. All measurements were performed at room temperature using a 25 mm diameter parallel plate geometry and maintaining a 0.5 mm gap from the rheometer platform. A transparent glass platform was used in the rheometer for placing the samples and allowing visible light irradiation. When recording the photocrosslinking kinetics, the visible light was irradiated on the thin prepolymer layer from the bottom to initiate the crosslinking as shown in Fig. 3(B). A visible light was projected 1 min after the commencement of the experiment and the exposure continued for a duration of 5 min. Oscillatory measurements were performed with 0.5 % shear strain and 1 Hz frequency. The storage and loss modulus for the photocrosslinking bioink were recorded simultaneously. After completing the exposure duration, angular frequency sweep was done in the range of 0.1–100 rad/s at constant 0.5 % shear strain to observe dynamic rheological properties. Next, the viscosity variation of the bioinks was recorded by varying the shear rates from 0.01 to 1000 s^−1^. The viscosity characterization of the bioinks was performed in absence of visible light irradiation.

### 2.8 Cell culture

Human hepatocellular carcinoma HepG2 cells were cultured utilizing high glucose Dulbecco's Modified Eagle Medium (DMEM) (Corning, Arizona, USA) supplemented with 10 % fetal bovine serum (FBS) (Corning, Arizona, USA) and 1 % penicillin/streptomycin (Cytiva, Marlborough, MA, USA). The cells were cultured in tissue culture flasks and maintained at 37 °C and 5 % CO_2_ in an incubator. For culturing the HepG2 cells, the growth medium was renewed every 3 days.

### 2.9 Cell-laden hydrogels viability assessment

For fabricating the cell-laden structures, the cells were seeded at a density of 2.6 × 10^2^ cells/mL in various bioinks and the crosslinked structures were cultured in the same growth medium renewing every 3 days. Cell viability analysis was conducted utilizing the LIVE/DEAD Viability/Cytotoxicity Kit (Biotium, Fremont, CA, USA). Following 3 washes with PBS, the encapsulated cells were subjected to staining for 30 min in the dark at room temperature using a PBS solution containing 0.5 μL mL^−1^ calcein AM and 2 μL mL^−1^ EthD. Subsequently, the samples underwent 3 additional washes with PBS to eliminate any remaining reagents. Fluorescence images were captured using an inverted fluorescence microscope, with Calcein AM fluorescence recorded in the FITC channel and EthD fluorescence in the TxRed channel.

### 2.10 Metabolic activity assay

Cellular mitochondrial metabolic activity was assessed by adding the oxidation-reduction indicator, tetrazolium hydroxide salt assay (XTT kit; Biotium, Fremont, CA, USA), at a ratio of 1/10 of the medium volume to evaluate cell proliferation. Following a 24-h exposure to XTT solution at 37 °C in a 5 % CO2 environment, 100 μl of medium from each well was transferred to a 96-well plate. The adsorption metabolite of resazurin, resorufin, was then measured using a spectral scanning plate reader with excitation at 450 nm.

### 2.11 Proliferation and morphological assay

To evaluate cell proliferation in hydrogel scaffolds containing either control or LdMA bioinks, cells were encapsulated and cultured for one month. A hydrogel prepolymer solution was prepared with 5 % w/v GelMA (5G) (used as the control) and 5 % w/v GelMA supplemented with 0.5 % LdMA (5G0.5LdMA). Cells were added at a density of 2.6 × 10^6^ cells ml^−1^ and evenly dispersed. The produced bioink served as the ink for the custom bioprinter described in an earlier report [38]. Multiple disks, each with a diameter of 4 mm and a thickness of 400 μm, were printed to ensure that at least one dimension fell in the range comparable to the critical limit for media diffusion. After crosslinking with visible light for 1 min in a custom-made DLP bioprinting system [38], the disks were transferred to a petri dish, washed with PBS, and incubated in fresh growth media at 37 °C with 5 % CO_2_. To assess cell morphology and proliferation, at least three disks per sample were stained with phalloidin (Cytoskeleton Inc., Denver, CO, USA) and DAPI (MilliporeSigma, Oakville, ON, Canada) for cytoskeleton and nuclei, respectively, at 7, 14, 21, and 28 days of culture. Briefly, samples were fixed with 4 % v/v paraformaldehyde for 90 min followed by permeabilizing with 0.5 % v/v Triton X-100 for 20 min, stained with phalloidin 568 for 90 min at room temperature. Then the samples were stained DAPI and mounted following by imaging, using an inverted fluorescence microscope equipped with DAPI and TRX channels.

### 2.12 D (bio)printing

The DLP-based bioprinter mentioned in Section 2.4 was used for 3D (bio)printing of different structures. The system consists of a visible light projector (as described in previous sections), a focusing system, and a z-axis motion platform. The design of each layer is projected in a black-and-white pattern, which is focused through a lens onto the dispensed prepolymer solution. The layer-by-layer printing method is illustrated in Fig. S10B and is based on single-layer patterning.

Briefly, a circular mold with a diameter of 20 mm and a height of 1 mm, with a glass base, was used. For each layer, to achieve a thickness of 50 μm, 20 μL of the prepolymer solution, with or without HepG2 cells (at a concentration of 2.6 × 10^2^ cells/mL), was dispensed into the mold. After a defined light irradiation time, the sample bed was lowered by 50 μm, followed by dispensing the next layer and photo-patterning. This layer-by-layer process was repeated until the entire 3D structure was fabricated. At the end of printing, the uncrosslinked prepolymer solution was removed, leaving only the 3D (bio)printed structure in the mold. The structures containing cells were cultured as described in Section 2.8. After one week of culture, the 3D structures underwent phalloidin/DAPI immunostaining as described in Section 2.11, and were imaged using a confocal microscope (Nikon Eclipse Ti, Nikon, Tokyo, Japan) to obtain high-resolution 3D and Z-stack images.

### 2.13 Immunostaining and fluorescent microscopy

Cell encapsulation and sampling was done according to the procedure mentioned in section 2.10. For immunofluorescence staining of Albumin, the manufacturer's protocol for antibodies was followed. Initially, 3D cultured HepG2 cells were rinsed with PBS to remove excess media absorbed by the hydrogels. Subsequently, the structures were fixed in 4 % paraformaldehyde in PBS for 90 min at room temperature. Following three washes with PBS containing 0.3 % Triton X-100 (washing solution), the cells were blocked with 1 % Bovine Serum Albumin (BSA) (Millipore Sigma, Oakville, ON, Canada) and 0.3 % Triton X-100 in PBS for 1 h to minimize nonspecific binding. After rinsing with the washing solution, cells were incubated with primary antibodies: rabbit anti-albumin antibody (ab207327; Abcam, Toronto, ON, Canada) diluted 1/500 in PBS at 4 °C overnight. Following incubation, 3D cultured cells were washed with the washing solution and then incubated with goat anti-rabbit secondary antibody Alexa Fluor 488 (ab150077; Abcam, Toronto, ON, Canada) for 2 h at room temperature. After three washes with PBS, the samples were transferred on a glass slide and mounted with the mounting solution containing DAPI. A coverslip was placed on top, and the samples were imaged using an inverted fluorescence microscope (ECHO revolve) equipped with DAPI and FITC channels.

### 2.14 Albumin secretion and metabolic analysis

To evaluate the liver-specific functions of cells within the hydrogel, secreted albumin levels were quantified using an Albumin ELISA kit (AFG Scientific, Northbrook, IL, USA), following the manufacturer's instructions for the same amount of sample from each group. Media was changed 24 h before sampling. Collected media samples were stored at −20 °C until further use and subsequently measured after appropriate 5 times dilution to ensure alignment with the kit's standard curve.

### 2.15 Drug-induced hepatotoxicity evaluation

For evaluating hepatotoxicity, acetaminophen (APAP) (Millipore Sigma, Oakville, ON, Canada) was utilized as the drug model in our in vitro study. The APAP stock solution was prepared by dissolving it in 100 % ethanol to achieve a 0.5M concentration. Seven consecutive concentrations were then prepared by diluting the concentrated stock solution in cell culture media. To obtain the dose-response curve, 2D HepG2 and 3D HepG2-laden structures in 5G and 5G0.5Ld were cultured for 10 days and then induced with drug-containing media. Cell viability was assessed using the XTT assay after 24 and 48 h, with fresh drug-containing medium renewed daily. Experiments were conducted in triplicate, and viability data were normalized against internal controls. The dose-response curve was generated as a semilogarithmic plot using a Variable Slope fitting model (GraphPad Prism ver 12.2.2, GraphPad Software, CA, USA). The half-maximal inhibitory concentrations (IC50) were calculated from the corresponding dose-response curves.

### 2.16 CYP1A2 expression level

CYP1A2, an enzyme belonging to the cytochrome P450 family, plays a crucial role in the metabolism of various endogenous and exogenous compounds, including drugs, toxins, and carcinogens. The CYP1A2 expression is commonly studied to understand liver function, drug metabolism, and toxicology. CYP1A2 expression level was quantified using a Human CYP1A2(Cytochrome P450 1A2) ELISA Kit (AFG Scientific, Northbrook, IL, USA), following the manufacturer's instructions. Collected media samples were stored at −20 °C until further measurement.

### 2.17 Statistical analysis

Quantitative data are reported as means ± standard deviations (SD), with inferential statistics (p-values) used for analysis. Statistical significance was determined using two-tailed t-tests and analysis of variance (ANOVA), with significance levels set at p < 0.05, p < 0.01, or p < 0.001. Analysis was conducted using GraphPad Prism ver 12.2.2 software.

## 3 Results and discussion

### 3.1 Decellularization assessment

In order to develop dECM-containing bioinks suitable for DLP bioprinting, Porcine fresh liver was used for liver decellularization and functionalization, combined with GelMA.

**Fig. 1:**
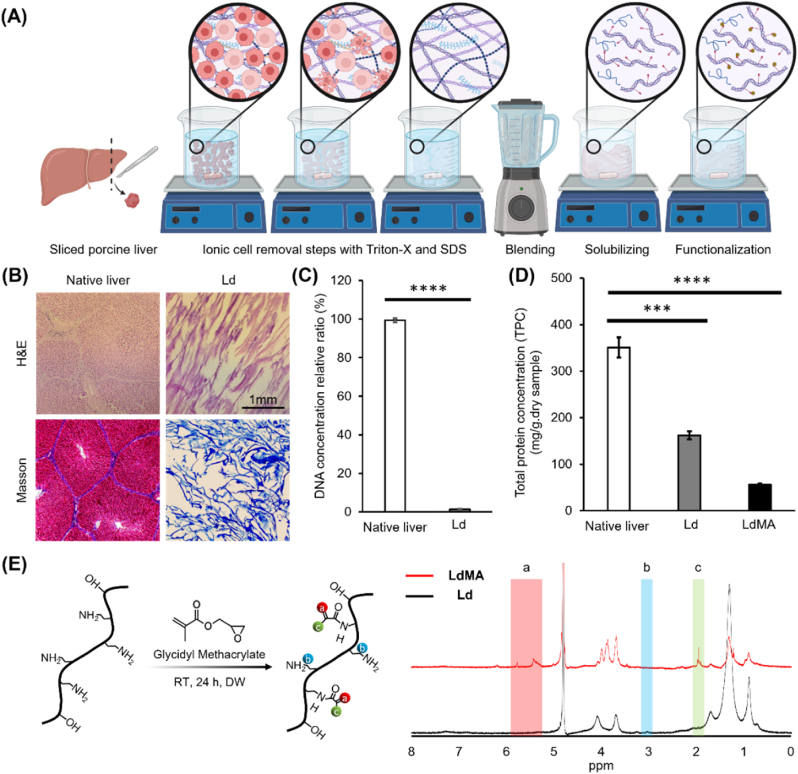
Liver dECM synthesis and methacrylation (LdMA synthesis) process and relevant characterization. (A) Schematic representing the washing steps, solubilizing, and functionalization steps. (B) H&E and Masson Trichrome staining of native liver tissue and decellularized liver tissue ECM. Biochemical characterization before and after decellularization (C) total DNA content evaluation. (D) Total protein concentration of native liver, Ld and LdMA. (E) The reaction mechanism of substitution of methacrylate groups on dECM backbone molecules, and degree of methacrylation characterization of LdMA using ^1^H NMR spectra of methacrylated and unmodified Ld. ∗p < 0.05, ∗∗p < 0.01, ∗∗∗p < 0.001 and ∗∗∗∗p < 0.0001.

To achieve this, the first step was to prepare an optimal bioactive liver-based material containing a maximum amount of ECM components while minimizing cellular components. The overall process is illustrated in Fig. 1(A). The decellularization process took three days to achieve optimal cell removal, and as shown in Fig. S1, the liver tissue became increasingly transparent as the process progressed. The decellularization was optimized to preserve key native liver ECM components, such as collagen strands, while ensuring complete cell removal. Finally, the Ld was lyophilized before undergoing the functionalization process. Histochemical staining was performed to visualize cell nuclear material and collagen content. As shown in Fig. 1(B), H&E stained section of native liver revealed intact liver tissue morphology showing the cells arrangement in hepatic lobules. In contrast, H&E staining of the dECM samples showed collapsed morphology of the lobules with no visible cell nuclei (stained in dark purple as shown in native liver tissue) proving successful cell removal. Additionally, Masson staining was performed to determine the presence of the collagen in obtained dECM. The result revealed that the main component remaining after decellularization steps is the collagen fibers as stained in blue. Furthermore, the methacryloyl functionalized dECM material (LdMA) underwent Masson's trichrome staining to assess the preservation of collagen fibers post-functionalization. Fig. S2 illustrates the prevalence of collagen fibers within the functionalized dECM sample. Nonetheless, when compared to Fig. 1(B), it is evident that the collagen fibers have become fragmented as a consequence of the solubilization process. Moreover, DAPI staining of both liver and dECM samples, as presented in Fig. S3, serves as additional evidence of successful cell removal. As a result, the Ld compound is anticipated to outperform as a bioactive biomaterials in promoting hepatic differentiation and enhancing hepatocyte-specific functions, owing to its liver-derived biochemical components.

To evaluate the decellularization efficacy quantitatively, we investigated the DNA and total protein content. It was observed that the DNA content in dECM samples dropped to less than 2.7 ± 0.31 % of the DNA content in native liver (normalized to native liver DNA content) as presented in Fig. 1(C). Although ionic treatments have high efficacy on cell removal, they are more aggressive than non-ionic treatments and will wash away more contents of ECM proteins. For investigating the influence of the washing and functionalization step on TPC. It has been shown that the TPC has decreased significantly from 350.96 ± 21.66 to 162.05 ± 8.58 mg/g of dry sample (Fig. 1(D)) which was predictable due to the usage of harsh ionic detergent (SDS) responsible for promoting protein erosion [33].

### 3.2 dECM functionalization and characterization

Widespread utilization of self-assembled ECM-based hydrogels is hindered by their slow and uncontrolled gelation process, as well as their poor mechanical properties that fail to mimic physiological conditions [40]. Present ECM hydrogel-based liver tissue models lack sufficient control over their physical characteristics [41,42], failing to adequately consider the relationship between hydrogel stiffness and liver physiology and pathology [43]. To address these challenges, researchers have explored the functionalization of natural polymers using photocurable components to rapidly produce hydrogels with controlled gelation properties [26,27,44]. By combining the benefits of natural polymers, such as biocompatibility and degradability, with the reproducible physicochemical properties achieved through chemical functionalization, these resulting hydrogels offer potential to create controlled, consistent, and relevant 3D tissue models [26,27]. However, only a limited number of studies have sought to modify liver ECM hydrogels in order to enhance their physicochemical properties [[45], [46], [47]]. In our study, due to presence of collagen as the abundant protein in synthesized dECM, amine (-NH_2_) amine functional groups on the backbone of collagen structure are suitable to be modified and could be substituted with methacrylate groups. However, collagen is required to undergo a solubilizing process in order to prepare the dECM for methacrylation process.

At this stage, pepsin has been utilized to enzymatically digest the matrix [48,49]. The action of pepsin involves cleaving peptide bonds at specific locations within the non-helical region of the collagen chain. This process effectively eliminates the non-helical immunogenic region of collagen, contributing to the removal of potentially immunogenic components from the matrix [50]. TPC of functionalized dECM was evaluated as it was the final product to be used in the bioink formulations. It is shown that the TPC has been reduced to 55.95 ± 2.65 mg/g of dry LdMA sample (Fig. 1(D)). To indicate the degree of substitution (DS) of methacrylate group, ^1^H NMR was done as presented in Fig. 1(E). The methacrylation of solubilized Ld led to the appearance of new peaks at 5.42, and 5.77 ppm and at 1.94 ppm that can be attributed to the new methacrylate and methacrylamide end groups on dECM macromolecules illustrated in Fig. 1(E). Integrating the peaks corresponding to internal reference groups indicates approximately 46 % DS. To elucidate the reaction occurring during the methacrylation process, a reaction pathway similar to that reported for GelMA is considered. The synthesis of GelMA is influenced by several parameters, including the gelatin source, the solvent used as the reaction medium, the reaction pH, the methacrylate group donor, the catalyst, and the reaction duration [51]. The reaction mechanism between GMA and gelatin is pH-dependent [52]. At higher pH, GMA undergoes hydrolysis, leading to the substitution of methacrylate groups primarily on the available primary amine (-NH_2_) groups of gelatin, with glycidol as a by-product. In contrast, at lower pH, the reaction follows an epoxide ring-opening mechanism, resulting in the grafting of a larger group without by-product formation. However, at lower pH, primary amine groups are largely protonated, which significantly reduces their reactivity. Carboxyl groups in gelatin can also act as grafting sites for GMA, though they are less reactive than amine groups at higher pH conditions [53]. Similarly for dECM, due to the presence of amine (-NH_2_) groups, and the reactioncondition which was at pH 8.5, primary amine sites were substituted. Accordingly, in Fig. 1(E), which illustrates the methacrylation chemistry of dECM, we depicted the primary amine (-NH_2_) substitution as the predominant reaction pathway, as it is the most likely mechanism under our experimental conditions.

### 3.3 Measurements of mechanical, swelling and degradation properties

**Fig. 2:**
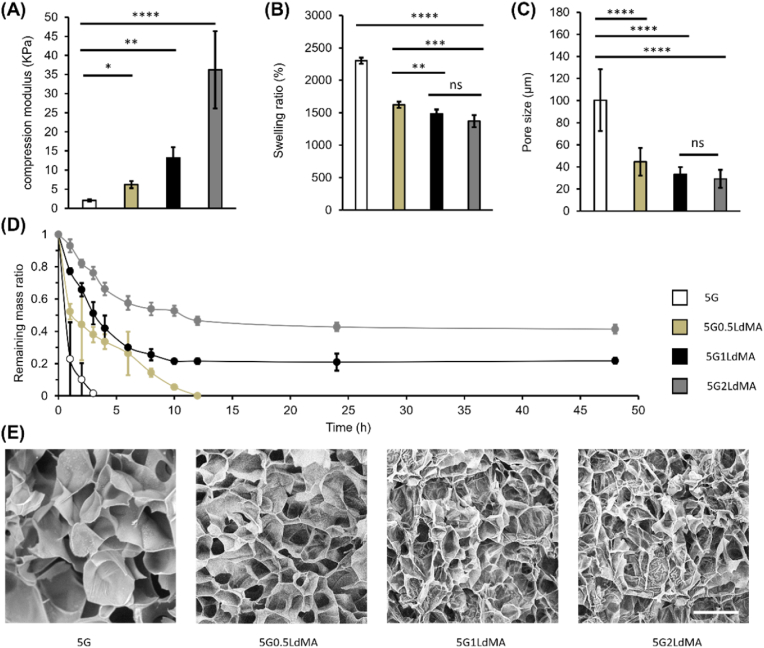
Mechanical and physical characterization of hybrid GelMA-LdMA hydrogels. (A) Compression modules of different compositions of hydrogels (n = 5). (B) Mass swelling ratio of different compositions of hydrogels (n = 5). (C) Pore size evaluation of the 3D microstructure of the hydrogels (n = 5). (D) Degradation characterization of the hydrogels under enzymatic degradation (n = 5), (E) Scanning Electron Microscopy images showing porous structure. Scale bar = 100 μm ∗p < 0.05, ∗∗p < 0.01, ∗∗∗p < 0.001 and ∗∗∗∗p < 0.0001.

It has been proven that the mechanical properties of the fabricated scaffold have significant influence on the cell fate and differentiation as well as on ECM remodeling [54,55]. Thus, we have measured the compression modulus of the hybrid GelMA-LdMA hydrogels to investigate the influence of the LdMA concentration. By adding LdMA to GelMA, an improvement in mechanical properties was expected due to higher degree of crosslinking due to an increase in the number of methacryloyl functional groups in the bioink solution. In the hybrid mode, it is not only the GelMA branches that make covalent connections during the photocrosslinking process, however, because of the presence of the alkenyl (C=C) groups on the LdMA backbone, they will participate in the crosslinking, resulting in increasing internal connectivity. As the concentration of LdMA increased from 0 to 2 % (w/v), a significant rise in the measured compression modulus was observed as shown in Fig. 2(A). The compression modulus of pristine GelMA was 2.06 ± 0.27 kPa and by adding 0.5 %, 1 % and 2 % of LdMA, the compression modulus ended up to 6.02 ± 0.95, 13.19 ± 2.84 and 36.21 ± 10.12 kPa, respectively, indicating an ability to increase the compression modulus up to 1658 % with respect to the control GelMA. This tunability in the mechanical properties makes the developed bioink a promising biomaterial for modeling different liver tissues. Various studies have investigated the mechanical properties of healthy liver and different stages of diseased or cancerous liver [56,57]. The elastic modulus of the native and healthy human liver is reported to be around 12 kPa [58] which indicates that the hybrid bioinks containing 0.5 % and 1 % (w/v) LdMA are promising biomaterials to model the healthy liver ECM. However, during different stages of fatty liver disease toward cancerous liver, the stiffness of the liver tissue would increase due to fat accumulation. In this case, higher concentrations of the LdMA leading to higher mechanical properties would be suitable to represent the desired scaffold stiffness in liver disease in vitro modeling. On the other hand, in vitro studies on HepG2 cell encapsulation for liver tissue modeling have demonstrated that scaffolds with a compression modulus of less than approximately 6 kPa promote optimal HepG2 aggregation and albumin secretion [19]. These findings suggest that both 5G and 5G0.5LdMA could be viable options in terms of mechanical properties for effective cell encapsulation.

**Fig. 3:**
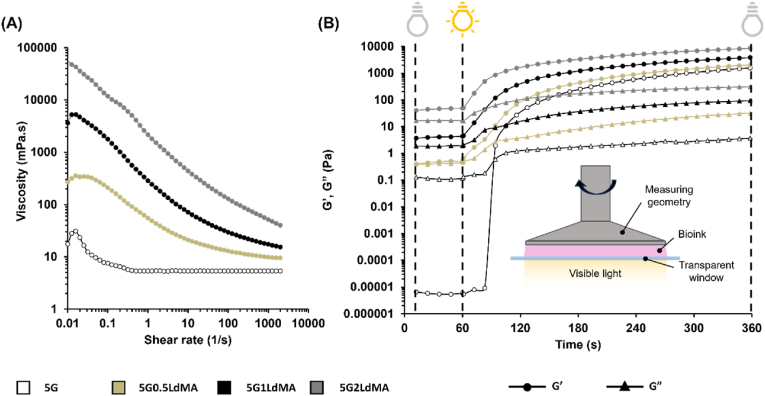
Dynamics rheological characterizations of GelMA and LdMA containing hydrogels. (A) Comparison of viscosity at different shear rates for different compositions of dECM-based hydrogels. Photocuring kinetics of GelMA and GelMA-LdMA hydrogels represented by (B) storage modulus and loss modulus recorded *in situ* crosslinking. For all *in situ* crosslinking characterizations, visible light irradiation started at t = 60 s and ended at t = 360 s.

The swelling behavior of hydrogels plays a crucial role in tissue engineering as it impacts several factors such as surface properties, and solute diffusion [59]. By measuring the swelling ratio of crosslinked hydrogel samples, we can gain insights into the crosslinking degree and the ability of the hydrogel to absorb water [60]. This capability is influenced by the pore size of the scaffold as well as the interactions between the solvent and polymer. Fig. 2(B) shows the swelling ratio data which by increasing the LdMA concentration illustrates a reciprocal trend in comparison to the mechanical properties. The control GelMA sample shows the highest swelling ratio equal to 2302.64 ± 47.59 %. Increasing the concentration of LdMA leads to a higher crosslinking density, resulting in a decrease in water retention capability. Consequently, hydrogels containing LdMA exhibit significantly lower swelling ratios as compared to pristine GelMA hydrogels, with the swelling ratio for 2 % LdMA reaching 1370.3 ± 94.6 %, which is approximately 1.6 times lower than the control GelMA. Interestingly, the reduction in swelling ratio between 1 % and 2 % LdMA is not significant, suggesting that the water retention capability reaches a saturation point. Consequently, further increasing the LdMA concentration has a relatively insignificant effect on the swelling ratio. This finding indicates that there may be a limit to the impact of LdMA concentration on the water retention properties of the hydrogels.

To further investigate the mechanical properties and swelling behavior of the hydrogels, their microstructures were examined using the SEM. The SEM images, as depicted in Fig. 2(E), reveal that the GelMA hydrogel contains pores with a size range of 70–130 μm (with an average of 100.34 ± 27.99 μm). This pore structure contributes to lower mechanical stiffness and a higher capacity to absorb water, as discussed earlier. However, with the addition of LdMA to the bioink, there was a significant reduction in pore size of the resulting hydrogels. The hydrogel containing 2 % LdMA exhibited a pore size of 29.13 ± 8.15 μm, which correlated with the highest compression modulus and the lowest swelling ratio. It is worth noting that the pore sizes observed in the LdMA-containing hydrogels did not show significant differences among themselves (Fig. 2(C)). This observation suggests that there was a saturation point in the decrease of pore size, which aligns with the trend in swelling ratio discussed earlier. In summary, the SEM analysis revealed that the addition of LdMA to the hydrogel formulation led to a reduction in pore size, resulting in improved mechanical stiffness and reduced swelling ratio. This indicates that the incorporation of LdMA influenced the microstructure of the hydrogel, leading to enhanced controllability of mechanical properties significantly without adversely influencing the swelling behavior.

Biodegradability has been a highly desirable characteristic in tissue engineering applications, particularly for hydrogels, as it indicates how long the scaffold can maintain its structural integrity. To investigate the degradation properties of GelMA-LdMA hydrogels, a series of experiments were conducted. Firstly, the dry weight of the samples was measured, and then they were rehydrated. Next, hydrogels with varying compositions were immersed in a collagenase solution and incubated at 37 °C. The weight of the hydrogels was recorded at different time intervals after lyophilization. As demonstrated in Fig. 2(D), the GelMA control sample was found to be completely degraded within 4 h. In contrast, the hybrid bioinks containing 1 % and 2 % LdMA exhibited a significantly slower degradation rate, preserving their structure for over 2 days. This outcome clearly indicates that the incorporation of LdMA extended the degradation time of GelMA. The differences in degradation rates between GelMA and the hybrid hydrogels can be attributed to two main factors. Firstly, the introduction of an additional methacrylated component to GelMA resulted in a higher degree of crosslinking. Consequently, during the degradation process, a greater number of chemical bonds needed to be broken down to cause structural collapse. Additionally, through swelling and microstructural evaluations, it was observed that higher concentrations of LdMA led to a reduction in pore size within the hydrogels. Consequently, during enzymatic degradation, the hydrogels absorbed a smaller amount of the solution, resulting in a decrease in the rate of degradation [61]. The ability to tune the degradation rate of these hybrid hydrogels makes them highly promising biomaterials for a wide range of applications, both *in vivo*, such as for implants, and also long-term in vitro application [62], including drug screening platforms.

Furthermore, structural analysis of samples containing dECM revealed the presence of wrinkles in the GelMA walls, as depicted in Fig. 2(E). These wrinkles became more pronounced with an increased concentration of LdMA, suggesting a direct correlation. This phenomenon can be attributed to the intrinsic structural characteristics of Ld and LdMA, as similar wrinkled structures are also observed in their SEM images (Fig. S4).

To validate the impact of dECM methacrylation on the physiological characteristics of the engineered hydrogels, control prepolymer samples incorporating unmodified dECM were prepared for comparison with these findings. The incorporation of various concentrations of solubilized dECM into 5 % GelMA resulted in an enhancement of mechanical properties, likely attributed to the increased overall concentration of the prepolymer solution, as illustrated in Fig. S5(A). Nonetheless, the compressive modulus of the resulting hydrogels were significantly less as compared to the samples containing LdMA. The integration of LdMA into GelMA, followed by light-induced crosslinking, caused additional chemical covalent bonds not only among GelMA chains but also between GelMA and LdMA fibers, unlike in samples with unmodified dECM where covalent bonding occurred solely among GelMA chains, excluding dECM fibers. Furthermore, in samples with unmodified dECM, the absence of any bonding between dECM fibers and the 3D structure of GelMA led to a significantly faster degradation by collagenase enzyme in the enzymatic degradation assay, particularly evident in the 5G0.5LdMA samples compared to both 5G and 5G0.5LdMA samples, as shown in Fig. S5(B).

### 3.4 Crosslinking time measurements and rheological characterization

The effective utilization of dECM-based bioink in DLP bioprinting relies on a material that features appropriately tuned rheological behavior. To achieve this, an assessment of both the viscosity and the photocrosslinking kinetics was conducted via rheological characterization. Illustrated in Fig. 3 are the viscosity and dynamic moduli for various formulations of dECM-based bioinks. As depicted in Fig. 3(A), the incorporation of LdMA into GelMA significantly increased the viscosity of the bioinks by a factor of 10–1000 times relative to the GelMA bioink, a consequence of the rich presence of collagen fibers in LdMA. It has been established in literature that the viscosity of the resin for optimal usage in light-based 3D printing should not surpass 5000 cPs [63,64]. Thus, this suggests that bioink formulations containing 2 % LdMA may not be ideally suited for light-based bioprinting applications. However, owing to the pronounced shear-thinning property exhibited by the 5G2LdMA sample, it emerges as a promising candidate for extrusion bioprinting, as verified by various studies exploring the extrudability of dECM-based bioinks in 3D bioprinting context [65,66].

Furthermore, the photorheological properties of the bioink were elucidated using a photorheological test wherein the storage and loss moduli of diverse hybrid bioinks were measured over 60 s in the dark to establish a baseline, followed by illumination with visible light to record changes. Fig. 3(B) displays the evolution of G′ and G″ during the photocuring process, offering insights into several critical aspects. The point at which the curves of G′ and G″ intersect is typically regarded as the material's gel point. In the case of the 5G sample, this gel point occurs approximately 30 s after the commencement of irradiation (Table S1). Conversely, in samples containing LdMA, the gel point was not observed within the photocuring timeframe, as indicated by the G′ values exceeding those of G″ prior to irradiation, likely due to a higher viscosity and overall gel concentration leading to a gel-like structure even before photocuring. Additionally, the period from the onset of exposure to the observable rise in G′ and the initiation of gelation, defined as gel time (GT), varies across samples [67]. For the 5G sample, gelation starts after a delay of about 20 s (Table S1), whereas, in samples with a high concentration of methacryloyl groups, the photo-crosslinking reaction initiates instantly upon light exposure, as evident from the absence of delay in the rheological data.

A crucial consideration in light-based printing is the development process i.e. the ability to easily separate the printed structure from the uncured bioink, which is pivotal for achieving high printing resolution. Optimal printing necessitates a printed structure with high stiffness and an uncured bioink with low viscosity. The logarithmic difference between the G′ values of the crosslinked and uncrosslinked states is quantified by ΔG’ which servs to articulate the rheological disparity between the printed hydrogel structure and the uncured bioink. Throughout the photocuring process, as the bioink undergoes increased crosslinking, the ΔG' value rises until a saturation is achieved in crosslinking, whereupon it stabilizes [67]. In this experiment, the samples were illuminated for 5 min to ensure that G′ reached its saturation point, signaling the completion of photocuring. The variations in mechanical properties previously discussed were also reflected in different final G′ and G″ values after crosslinking, which increased with increasing LdMA concentration, thereby elevating ΔG' (Table S1). Moreover, literature indicates that achieving the optimal crosslinking density during photo-crosslinking is indicated by 75 %–80 % of the ΔG’ logarithmic value, defining this range as the printable window [67]. Table S1 presents the printable windows for various bioinks, illustrating that an increase in LdMA concentration shortens the crosslinking time, attributed to a higher concentration of methacryloyl groups.

Lastly, sustaining the printed structure's shape requires greater elasticity than viscosity. A larger difference between G′ and G″ values imply a better shape retention. The term ΔM denotes the logarithmic difference between the G′ and G″ values following full exposure during photocuring [67]. Table S1 details ΔM values for different bioinks, revealing that as LdMA concentration rises, so does the degree of methacrylation, yielding a stiffer structure and, consequently, a larger ΔM value.

To explore the viscoelastic properties of crosslinked hydrogels, Fig. S6 presents the comparison between the elastic modulus and the viscous modulus of photocrosslinked hydrogels across various angular frequencies. Throughout the entire range of angular frequencies, the G′ values for all hydrogel formulations consistently exceeded those of G″, indicating a predominantly gel-like viscoelastic behavior. For hydrogels incorporating LdMA, the profiles of G′ and G″ moduli appeared as parallel straight lines across the majority of the angular frequency spectrum, with no points of intersection observed, suggesting uniform viscoelastic characteristics. Conversely, the 5G sample exhibited a deviation from this pattern, with G′ and G″ not maintaining uniformity throughout the angular frequency range, reflecting its unstable structural integrity [68,69].

### 3.5 Printability evaluation

As discussed in previous studies, an optimal photocurable bioink must meet specific criteria, including rapid photo-crosslinking, high crosslinking density, and enhanced fluidity [67]. Firstly, a high crosslinking rate is imperative to accelerate curing, thus reducing printing time and enhancing printing resolution. Secondly, maintaining a high crosslinking density is essential to impart adequate stiffness, thereby preventing deformation. Lastly, low viscosity is critical for improving printing efficiency, facilitating rapid flow of uncured bioinks into gaps and spaces, and simplifying the removal of uncrosslinked ink to achieve the desired final shape of hydrogels [67].

**Fig. 4:**
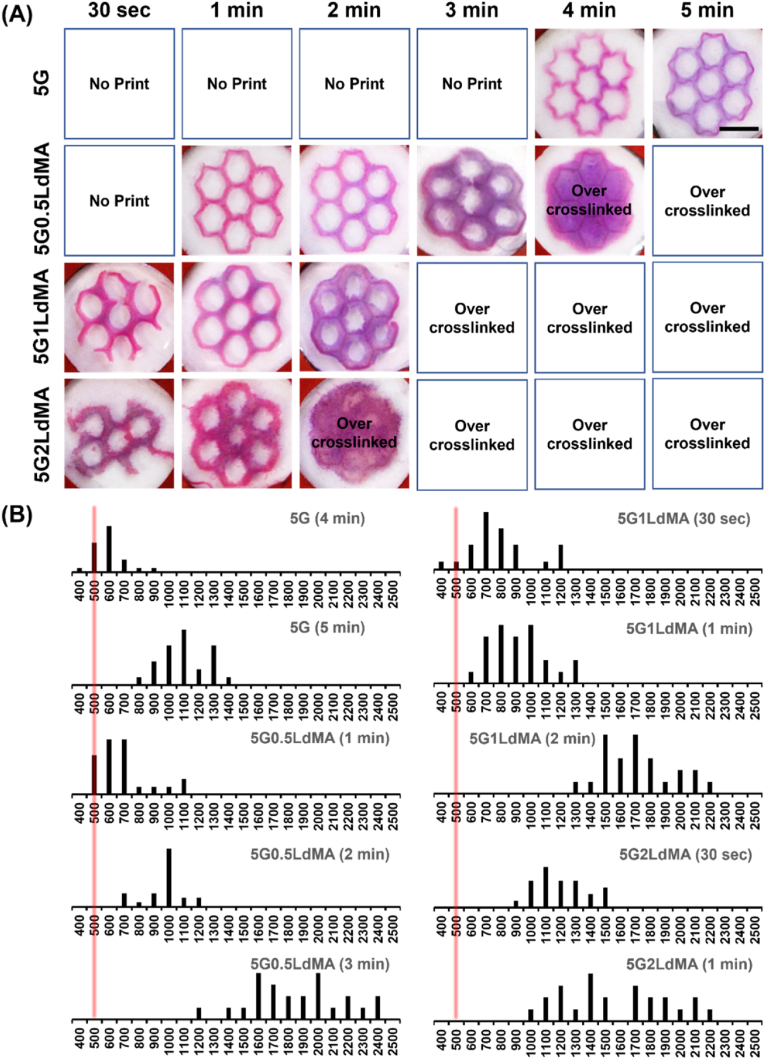
DLP printability evaluation. (A) Photo-patterning of different compositions of LdMA containing inks under various exposure times. The fabricated scaffolds were stained with red dye for better visualization. Scale bar = 5 mm. (B) Printed line thickness distribution of different constructed patterns.

To assess the printability of various compositions of GelMA and LdMA inks, a honeycomb pattern featuring seven hexagons was subjected to different durations of visible light irradiation for crosslinking. Fig. 4(A) illustrates the printed structures corresponding to these compositions. The control sample, 5G, required a minimum irradiation time of 4 min for crosslinking using a custom-made bioprinting system [38]. Subsequently, exposure for 4 min yielded a stable structure with undulating and wavy walls, while a longer exposure of 5 min resulted in increased stiffness of the walls. Conversely, the presence of 0.5 % LdMA led to the formation of a stable and rigid structure after just 1 min of visible light exposure. However, after 3 min of photocrosslinking, significant over-crosslinking occurred, leading to noticeable reduction in the size of holes within the hexagons. Similarly, for the 5G1LdMA sample, a 30 s exposure produced a moderately stable structure with distinguishable but broken branches, while a 1 min exposure yielded a firm and stable structure. However, exposure exceeding 1 min led to over-crosslinking of the 5G1LdMA ink. Furthermore, increasing the LdMA content to 2 % resulted in incomplete patterning even after a 30 s exposure, with over-crosslinking observed after 1 min of irradiation. Increasing the LdMA content enhanced the speed of crosslinking by enhancing the methacrylol groups, thereby enabling the formation of stable structures with reduced exposure time. Overall, the dECM-based bioink fulfilled the criteria for rapid photocrosslinking and achieving high crosslinking density.

In the evaluation of suitable inks for light-based printing, fluidity emerges as a crucial third criterion as mentioned in previous section. Rheological examinations, as discussed in the preceding section, have demonstrated that all examined inks, with the exception of the 5G2LdMA ink, fall within this acceptable viscosity range. Notably, the viscosity of the 5G1LdMA ink approaches the upper limit of this range. Despite the fact that both the 5G1LdMA and 5G2LdMA inks could be crosslinked within a 30-s timeframe, their relatively higher viscosity presented substantial difficulties in the DLP printing process. The process of washing away uncrosslinked ink proved to be particularly problematic for these ink compositions, leading to the breakage of printed structures post-printing.

Nevertheless, the photo patterning duration observed diverges from the optimal crosslinking time outlined in the prior section (Table S2). This difference can be attributed to the varying sensitivities of rheology and photo patterning investigations towards alterations in the crosslinking density and the architecture of the network. Rheological analysis is concerned with the assessment of the overall mechanical properties of the bulk material, in contrast to photo patterning studies which might prioritize the crosslinking within specific localized areas. Moreover, photo patterning could involve the utilization of ink layers that are thinner or prepared under different conditions. The thickness of the ink layer, its shape, or the manner in which it is exposed to light are factors that can significantly impact the kinetics of crosslinking, potentially resulting in variations in the determination of optimal crosslinking times across these methodologies.

Resolution is a critical factor in light-based bioprinting that needs careful consideration. The resolution of printing is fundamentally influenced by the rate and density of photo-crosslinking, which are, in turn, dictated by the production rate and concentration of free radicals in radical mediated crosslinking inks like methacrylates [67]. A rapid photo-crosslinking process minimizes the opportunity for intermediate chemical species like monomers, oligomers or free radicals to diffuse from the irradiated area, thereby ensuring more precise control over polymerization sites. This precision facilitates the creation of finer features and more defined boundaries, leading to an enhancement in resolution [70,71].

To assess the printing resolution, two key parameters are examined: the thickness of printed lines and the clarity of the edges of hexagonal structures which is reported in Fig. 4(B). In terms of line thickness, the control sample, designated as 5G, displayed a branch thickness of approximately 1054.16 ± 159.32 μm following a 5-min crosslinking period. In contrast, the complete structure printed using 5G0.5LdMA ink after just 1 min of photo-patterning exhibited line thicknesses of 723.88 ± 169.21 μm, which is comparable to the thickness of lines printed with 5G ink over 4 min, measuring around 556 ± 102.19 μm, although with wavy appearance and poor structural stability. Additionally, the 5G ink yielded relatively sharp outer edges after 5 min, despite the areas within the hexagons appearing somewhat circular. Conversely, structures printed with 5G0.5LdMA ink after 1 min of crosslinking showcased sharpness in both outer and most inner edges. This comparison indicates that the incorporation of 0.5 % LdMA into 5 % GelMA not only significantly reduces the crosslinking time by a factor of 5 but also enhances the resolution. It is important to note, however, that adding LdMA to GelMA results in reduced transparency, leading to increased light scattering and, consequently, a potential decrease in printing resolution [72,73]. This phenomenon might explain the observed increase in branch thickness in structures printed with 5G1LdMA ink after 1 min of crosslinking, which was approximately 868 ± 181.48 μm. Despite these challenges, the outer edges of hexagons printed in this experiment remained sharply defined and well-formed.

Finally, for the 5G2LdMA ink, the substantial decrease in transparency coupled with a significant increase in viscosity led to an increase in line thickness to 1143.21 ± 182.88 μm and 1518 ± 349.43 μm after 30 s and 1 min of photo-patterning, respectively. Moreover, the thicker lines resulted in the absence of sharp edges, rendering this ink less suitable for light-based printing applications due to its compromised resolution.

In addition to photo-patterning studies, 3D structures were also printed using layer-by-layer DLP printing of 5G0.5LdMA as shown in Fig. S11C. The results indicate that the proposed bioink is capable of printing various structures with different shapes and thicknesses, ranging from 0.5 mm to 3 mm, with high printing accuracy as shown in the brightfield images (Fig. S10D).

### 3.6 Viability evaluation and metabolic assay

**Fig. 5:**
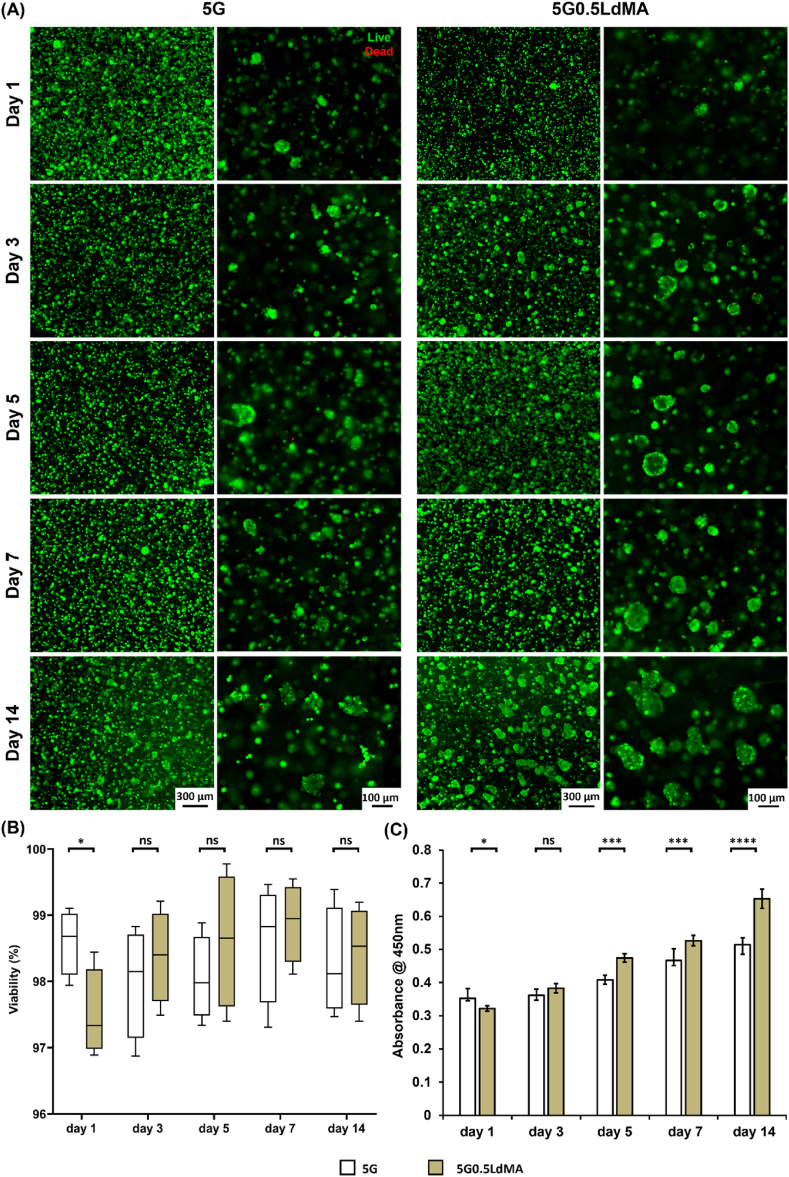
Biocompatibility and metabolic characterization of LdMA containing hydrogels. (A) Representative images showing live and dead HepG2 cells encapsulated in the hydrogels over 14 days of culture. (B) Cell viability evaluation calculated by the number of the live and dead cells (n = 3). (C) XTT assay showing HepG2 cells metabolism and proliferation within 14 days of culturing cells in 3D hydrogel structures (n = 5). ∗p < 0.05, ∗∗p < 0.01, ∗∗∗p < 0.001 and ∗∗∗∗p < 0.0001.

To explore the viability of HepG2 cells encapsulated in both control samples and those containing LdMA, bioprinted constructs were formed by photocrosslinking and were subsequently cultured for approximately one month. During various stages of the culture period up to 14 days, live/dead staining was performed on the scaffolds to assess cell viability. The imagery presented in Fig. 5(A) illustrates that, across all time points and in GelMA matrices with and without LdMA, the majority of cells not only endured the printing process but also proliferated within the scaffolds. Previous research has noted that HepG2 cells tend to aggregate into clusters as time progresses, with these clusters growing in size and eventually merging to form larger colonies [[74], [75], [76]]. A distinct difference was observed between cells in the 5G and the 5G0.5LdMA scaffolds, with cells in the latter tending to cluster more rapidly than in the control. Given the cellular tendency for cluster formation, traditional individual cell counting methods were inadequate for viability assessment. Instead, cell viability was quantified based on counting the number of pixels corresponding to live and dead cells. Throughout the 14 days culture period, the viability of HepG2 cells remained above 95 % in both scaffold types, indicating the biocompatibility of both GelMA and GelMA enhanced with LdMA matrices (Fig. 5(B)). Initially, cell growth was more pronounced within the 5G scaffold compared to its LdMA-enhanced counterpart, likely due to the former's lower mechanical properties and higher swelling ratio [77]. However, from day 3 onwards, the incorporation of LdMA rendered the scaffold more alike to native liver tissue, thereby fostering enhanced proliferation in the 5G0.5LdMA samples. From days 3, 5, 7, and 14 of culture, cell clusters in the 5G0.5LdMA samples exhibited accelerated growth in both size and quantity, as demonstrated by live/dead imaging in Fig. 3(A). In addition to visual assessments, an XTT assay was conducted to measure the metabolic activity of the cells to further quantify cell proliferation (Fig. 5(C)). Initially, the 5G scaffold housed more cells compared to the 5G0.5LdMA sample, resulting in higher absorbance readings due to the control hydrogel's lower stiffness. Nevertheless, as HepG2 cells within the LdMA-enriched scaffold proliferated from the third day of culture, they experienced an environment more closely resembling the native liver ECM niche, which significantly enhanced their metabolic activity compared to those in the 5G scaffold. This suggests a promising capability of LdMA containing scaffolds to support liver tissue regeneration.

### 3.7 Cell proliferation and morphology assessment

**Fig. 6:**
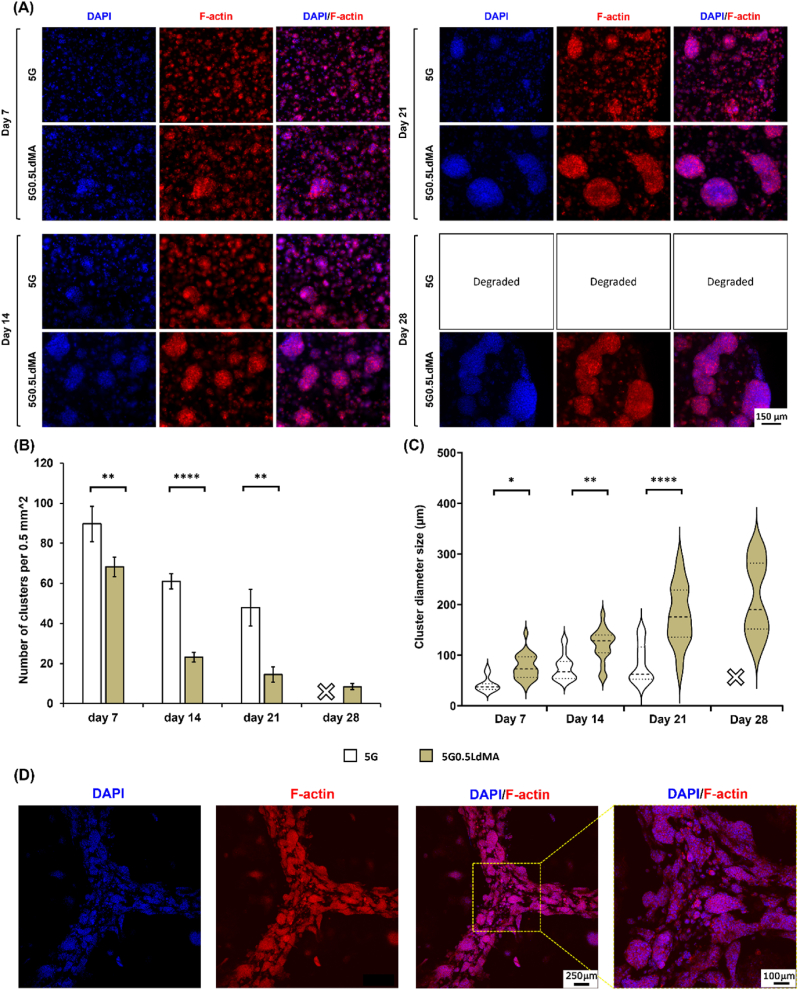
Evaluating cell growth, proliferation and morphology over 28 days of culture. (A) Fluorescent images showing HepG2 cells morphology during the culture period with phalloidin stained F-actin (red) and DAPI stained nuclei (blue) and the merged pictures. (B) HepG2 cell cluster number per unit of area on different days of the culture (n = 5). (C) HepG2 cluster size distribution over different days of the culture (n = 5). (D) Cell laden 3D bioprinted structures after 7 days of culture confocal imaging. ∗p < 0.05, ∗∗p < 0.01, ∗∗∗p < 0.001 and ∗∗∗∗p < 0.0001.

To examine the cellular morphology and organizational patterns within the bioprinted hydrogel scaffolds, staining of the cytoskeleton and nuclei was conducted at various culture intervals, as depicted in Fig. 6. GelMA was used as the base hydrogel matrix owing to its cytocompatibility and superior photo-patterning capability. It was further enriched with LdMA due to being abundant in intricate extracellular matrix proteins and growth factors.

The findings demonstrated a progressive development of HepG2 cells, evolving from individual cells into cohesive clusters, and ultimately forming multicellular spheroids, similar to previous studies [76,78]. These spheroids remained viable and stable for the entire duration of the study while exhibiting uniform distribution across the 3D scaffolds. In both the control and the LdMA containing hydrogels, cluster formation was evident by day 7 (Fig. 6), with Fig. 6(B) and **(C)** showcasing the number of clusters per unit imaged area and the average cluster size, respectively. On day 7, the LdMA enriched samples (5G0.5LdMA) not only exhibited fewer clusters than the control samples (5G) but also displayed significant differences in cluster sizes, 76.86 ± 24.73 μm for 5G0.5LdMA samples versus 41.76 ± 14.11 μm for the control, indicating more pronounced cell proliferation in the LdMA samples even within the first week of culture. By day 14, clusters in the LdMA-supplemented GelMA were notably larger and more distinct compared to those in 5G, emphasizing the enhanced cellular proliferation.

Clusters in the presence of LdMA demonstrated a more coherent and structured arrangement in contrast to the clusters formed in the GelMA-only hydrogels. These clusters were characterized by a well-defined cytoskeleton and multicellular aggregates that expanded in size as the culture period progressed. By the third week of culture (day 21), the average cluster sizes in the 5G and 5G0.5LdMA samples had grown to 179.27 ± 59.87 μm and 82.98 ± 34.00 μm, respectively (Fig. 6(C)). Additionally, a decreasing trend in cluster number was observed for both sample types implying merging of clusters.

As highlighted in the scaffold degradation study, the 5G samples exhibited the highest rate of degradation and, compounded by the cellular presence, did not maintain structural integrity until day 28 of culture and were completely degraded. On the other hand, Fig. S7 illustrates that in the 5G0.5LdMA samples, cell clusters began merging from day 21, leading to the formation of larger cell aggregates by day 28 and occupying significant portions of the hydrogel structure.

In summary, the incorporation of LdMA into the hydrogel scaffolds significantly promoted HepG2 cell spreading and proliferation. This enhancement is likely facilitated by the extracellular matrix proteins and growth factors inherent to the dECM component within the scaffold, and potentially through modifications to the mechanical strength and architecture of the GelMA network [78].

By observing the advantages of LdMA in promoting HepG2 cell proliferation and the formation of more mature microstructures compared to pristine GelMA, the 5G0.5LdMA ink was used for 3D bioprinting of hexagonal structures. Fig. 6(D) shows that HepG2 cells encapsulated in the bioprinted structures proliferated even more than in photo-patterned discs over the same 7-day culture period, forming mature in vitro liver scaffolds. This enhanced growth may be attributed to the structure thickness being below the critical nutrient diffusion limit. Additionally, Video S1 presents the 3D structure of the bioprinted constructs.

Supplementary data related to this article can be found online at https://doi.org/10.1016/j.mtbio.2025.101824

The following is the Supplementary data related to this article:

Video 1 Video 1

### 3.8 Albumin secretion analysis and immunostaining

**Fig. 7:**
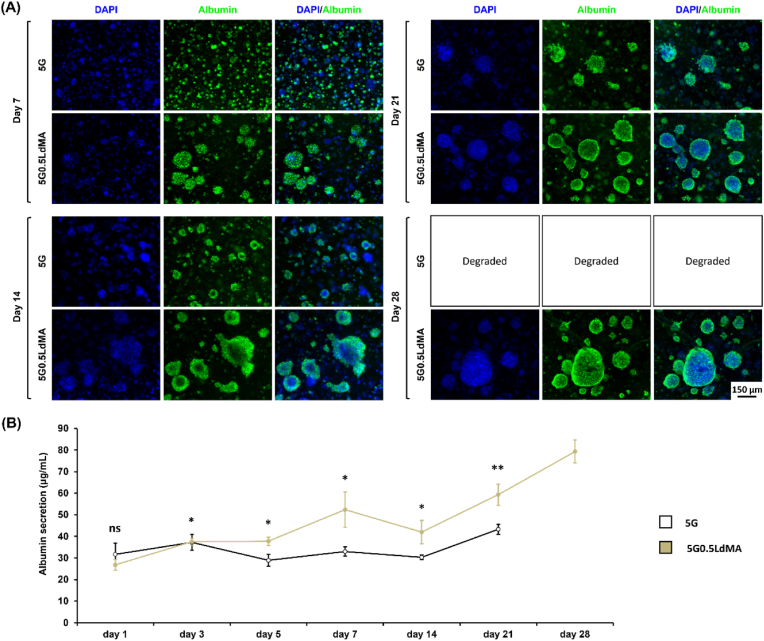
Liver functionality evaluation of 3D cultured HepG2 within hydrogels. (A) Albumin secretion activity visualized through immunostaining at different days of the culture. (B) Albumin production by HepG2 over the culture period in pristine and LdMA containing hydrogels (n = 5). ∗p < 0.05, ∗∗p < 0.01, ∗∗∗p < 0.001 and ∗∗∗∗p < 0.0001**.**

To verify the preservation of HepG2 cell functionality within the hydrogel scaffolds, the production of albumin - a marker of hepatocyte function - was assessed through immunostaining at various culture intervals (days 7, 14, 21, and 28) [5,74]. Evidence of albumin secretion was detected at all evaluated time points in both type of hydrogels (5G and 5G0.5LdMA), affirming the functional capacity of the bioprinted HepG2 cells within the hydrogels. Notably, HepG2 cells exhibited a marked increase in albumin production over time, particularly when cultured in hydrogels enriched with LdMA, in comparison to those in the control hydrogel. As illustrated in Fig. 7(A), albumin secretion was predominantly localized within HepG2 cell clusters formed in the 3D scaffolds. Despite both sample types exhibiting localized albumin production within cell aggregates throughout the culture duration, the control samples (5G) displayed a lack of significant albumin secretion in some cell clusters after the first and second weeks of culture. On the other hand, the LdMA-enhanced hydrogels (5G0.5LdMA) consistently showed significant albumin secretion across the majority of matured clusters. A combined assessment of cell morphology and immunostaining for albumin is presented in Fig. S8.

In addition to immunostaining, albumin secretion by HepG2 cells within the hydrogels was quantified using an ELISA kit, with the findings summarized in Fig. 7(B). From day 3 onwards, the 5G0.5LdMA samples demonstrated significantly higher albumin secretion levels compared to the control samples, attributed to the enhanced cell proliferation observed in the LdMA-supplemented hydrogels. Specifically, albumin secretion levels increased from 31.69 ± 5.25 to 43.31 ± 2.25 μg/mL (representing a 1.4-fold change) for the 5G after day 21 and from 26.79 ± 2.41 to 79.28 ± 5.31 μg/mL (indicating a 2.9-fold change) for 5G0.5LdMA after day 28 of culture. The albumin secretion for 5G samples was not quantified on day 28 as these samples were completely degraded. This progression underscores the significant impact of LdMA incorporation on enhancing HepG2 cell functionality within the scaffold environment.

### 3.9 Histology assessment

The objective of this characterization is to shed light on the histological variances observed in HepG2 cells cultured on GelMA scaffolds, both with and without the addition of LdMA. Utilizing a consistent H&E staining protocol across both sets of samples, we aimed to highlight the differences in cellular morphology and the interplay with the matrix. In sections stained with H&E, HepG2 cells within the pure GelMA scaffolds (Fig. S9) display the expected cytoplasmic staining characteristics, showcasing nuclei distinctly bordered by the stark blue of the hematoxylin stain and a cytoplasm that stands out in a contrasting lighter pink eosin stain. In contrast, HepG2 cells within the GelMA scaffolds were enriched with LdMA (Fig. S9) are marked by a significantly more intense cytoplasmic staining. This suggests a unique interaction with the eosin dye, which is an acidic stain that preferentially associates with basic cellular components, predominantly the protein elements present in the cytoplasm. Verifying with literature, the ECM is recognized for its imperative influence over cellular function and the modulation of gene expression, which consecutively impacts the synthesis of proteins within the cytoplasm [[79], [80], [81]]. Consequently, the deeper staining of the HepG2 cells’ cytoplasm in the LdMA-enhanced sample could be reflective of an increased synthesis of cytoplasmic proteins, potentially due to advanced cellular maturation processes.

Furthermore, the presence of unoccupied spaces within the clusters of cells observed in the GelMA-only sample, as opposed to the densely populated cellular arrangement seen in the 5G0.5LdMA sample, might stem from different causes. These include, but are not limited to, heightened cellular proliferation and migration, potentially spurred by the growth factors and other signaling molecules contained within the dECM-supplemented sample, which lead to diminished unoccupied volume between the cellular clusters.

Additionally, Masson's trichrome staining was employed to ascertain the presence of collagen fibers within the scaffolds utilized for HepG2 culture. In the 5G sample, as illustrated in Fig. S9, an absence of collagen fibers is noted, correlating with the initial lack of collagen supplement. In contrast, the 5G0.5LdMA sample reveals that the collagen fibers, intrinsic to the LdMA, have been retained within the structure after one month of culture, likely due to covalent linkages formed with the GelMA's backbone and slower degradation rates. This finding underscores the sustained incorporation of LdMA within the GelMA scaffold as being advantageous for the prolonged cultivation of liver cells.

### 3.10 Hepatotoxicity testing of cell-laden structures and 2D cells

**Fig. 8:**
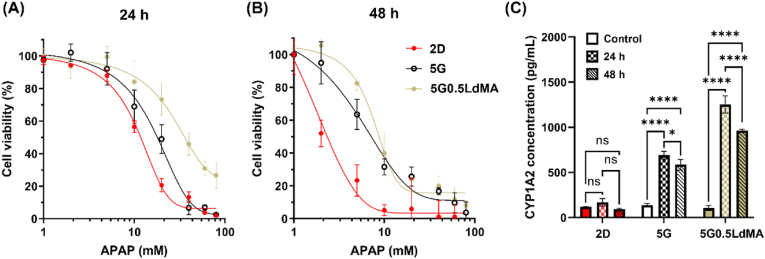
APAP-induced hepatotoxicity of the hepatic tissue drug testing models. (A) Dose-response curves after 24 h treatment of APAP. (B) Dose-response curves after 48 h of treatment with APAP. (C) Expression of CYP1A2 measured at the 2 time points under the APAP exposure of IC50 concentrations (n = 3). ∗p < 0.05, ∗∗p < 0.01, ∗∗∗p < 0.001 and ∗∗∗∗p < 0.0001.

An overdose of APAP is the leading cause of acute liver failure in numerous countries. APAP triggers liver damage by inducing mitochondrial dysfunction, leading to hepatocyte necrosis [82]. To investigate the potential of dECM-containing hydrogels as liver in vitro models for drug screening applications, their response to APAP as a drug model was studied. To determine the ideal sublethal drug concentration for toxicity evaluation, a dose-response curve on 2D-cultured HepG2 and cell-laden hydrogels was generated using the XTT assay, with results after 24 h and 48 h shown in Fig. 8(A) and **(B)**. After 24 h of treatment with varying APAP concentrations, the IC50 values for the 2D, 5G, and 5G0.5LdMA models were 12.55, 19.20, and 44.91 mM, respectively. After 48 h, the IC50 values decreased to 1.88, 6.13, and 9.63 mM, respectively (Table S3). In both time points, the 5G0.5LdMA sample showed greater resistance to APAP, followed by 5G, and thus IC50 concentrations were used for further drug metabolism-related enzyme CYP1A2 metabolic activity evaluation.

As illustrated in Fig. 8(C), after 24 h and 48 h of treatment, the CYP1A2 level was significantly upregulated in the 3D constructs compared to the 2D models. The 5G0.5LdMA sample demonstrated stronger resistance to APAP than 5G, likely due to larger and more mature cell spheroids formed in the 5G0.5LdMA sample [83]. However, after 48 h of treatment, the decrease in cell viability of the 5G0.5LdMA sample was more pronounced than in 5G (Fig. 8(B)), with the IC50 value dropping by 79 % in 5G0.5LdMA compared to 68 % in 5G. This could be attributed to the pronounced upregulation of CYP1A2, which increases drug sensitivity. CYP1A2 plays a role in the metabolism of APAP, converting it into the toxic intermediate N-acetyl-p-benzoquinone imine (NAPQI) [84]. Additionally, as shown in Fig. 8(C), when HepG2 cells in hydrogels are exposed to APAP, CYP1A2 upregulation may occur as part of the cells' attempt to metabolize the drug more efficiently. This upregulation is a cellular response aimed at enhancing the metabolism of xenobiotics like APAP, leading to increased production of the active enzyme. It is evident that CYP1A2 expression in the 5G0.5LdMA sample is higher than in 5G. The increased sensitivity to APAP and the elevated expression of the CYP1A2 enzyme from 0 to 24 h demonstrate the enhanced drug testing performance of our proposed dECM-based constructs, outperforming traditional 2D culture systems and control 3D structures [85].

During prolonged drug treatment, CYP1A2 expression decreased after 48 h compared to 24 h. Various studies have consistently shown significantly decreased levels of phase I CYP mRNA over time [29], which is due to APAP-induced hepatotoxicity leading to oxidative stress, mitochondrial dysfunction, and ultimately, cell damage, resulting in decreased expression and function of CYP enzymes, including CYP1A2 [86].

In this study, HepG2 cells were used as a proof-of-concept model to evaluate the designed bioink. While HepG2 cells provide a convenient and well-characterized system for initial assessments, we acknowledge that they do not fully replicate the complexity of a physiologically relevant in vitro liver model for clinical translation. Moving forward, we plan to investigate the bioink's suitability using primary human hepatocytes or iPSC-derived hepatocytes to further assess its potential for advanced liver tissue engineering applications.

## 4 Conclusion

The challenges posed by the inadequate mechanical and physical properties, along with the limited printability of dECM containing bioinks, have been significant barriers to their application in 3D bioprinting technologies. To address these issues, our research introduces a novel liver dECM-based bioink, created by methacrylated Ld. Grafting LdMA to GelMA enhanced the hydrogel's suitability for bioprinting and long-term cell culture. Additionally, LdMA prevented phase separation, ensuring homogeneous distribution within the GelMA network, unlike physically blended dECM, which may separate due to differences in solubility and viscosity. These advantages highlight LdMA's superiority in maintaining scaffold integrity and functionality over time. The presented novel LdMA-containing bioinks demonstrated rapid crosslinking under visible light and tunable physicochemical properties of the resulting hybrid hydrogels. Extensive characterizations confirmed these new bioinks' significant improved mechanical and rheological properties, as well as their capability for precise photo-patterning. Furthermore, in vitro experiments using HepG2 cells revealed outstanding biocompatibility, with evidence of enhanced cell proliferation and replication of functionality of HepG2 *in vivo* when compared to control samples. Looking ahead, the LdMA-containing bioinks developed in this study hold promising potential as a sophisticated resource for regenerative medicine and drug screening endeavors.

## CRediT authorship contribution statement

**Nima Tabatabaei Rezaei:** Writing – review & editing, Writing – original draft, Visualization, Validation, Software, Resources, Project administration, Methodology, Investigation, Formal analysis, Data curation, Conceptualization. **Hitendra Kumar:** Writing – review & editing, Methodology. **Hongqun Liu:** Writing – review & editing. **Ashna Rajeev:** Writing – review & editing. **Giovanniantonio Natale:** Writing – review & editing. **Samuel S. Lee:** Writing – review & editing. **Simon S. Park:** Writing – review & editing. **Keekyoung Kim:** Writing – review & editing, Validation, Supervision, Project administration, Methodology, Investigation, Funding acquisition, Formal analysis, Conceptualization.

## Declaration of competing interest

The authors declare that they have no known competing financial interests or personal relationships that could have appeared to influence the work reported in this paper.

## Data Availability

Data will be made available on request.
