# Supplementary material for: Bioprinting of hepatic tissue model using photocrosslinkable dECM-containing composite hydrogel
Source: Mater Today Bio. 2025 May 3;32:101824. doi: 10.1016/j.mtbio.2025.101824 (PMC12141563; doi:10.1016/j.mtbio.2025.101824)
Supplement: Multimedia component 1 [file mmc1.docx]

**Supporting Information**

### **Bioprinting of Hepatic Tissue Model Using Photocrosslinkable dECM-containing Composite Hydrogel**

*Nima Tabatabaei Rezaei^1^, Hitendra Kumar^2^, Hongqun Liu^3^, Ashna Rajeev^4^, Giovanniantonio Natale^5^, Samuel S. Lee^3^, Simon S. Park^1^,*

*and Keekyoung Kim^1, 6, *^*

^1^Department of Mechanical and Manufacturing Engineering, University of Calgary, Calgary, Alberta, T2N 1N4, Canada

^2^Department of Biosciences and Biomedical Engineering, Indian Institute of Technology Indore, Indore, Madhya Pradesh 453552, India

^3^Liver Unit, Cumming School of Medicine, University of Calgary, Calgary, Alberta, T2N 1N4, Canada

^4^Department of Chemical Engineering, University of Waterloo, Waterloo, Ontario, N2L 3G1, Canada

^5^Department of Chemical & Petroleum Engineering, Schulich School of Engineering, University of Calgary, Calgary, Alberta, T2N 1N4, Canada

^6^Department of Biomedical Engineering, University of Calgary, Calgary, Alberta, T2N 1N4, Canada

^*^Corresponding author: Dr. Keekyoung Kim, email: [keekyoung.kim@ucalgary.ca](mailto:keekyoung.kim@ucalgary.ca)


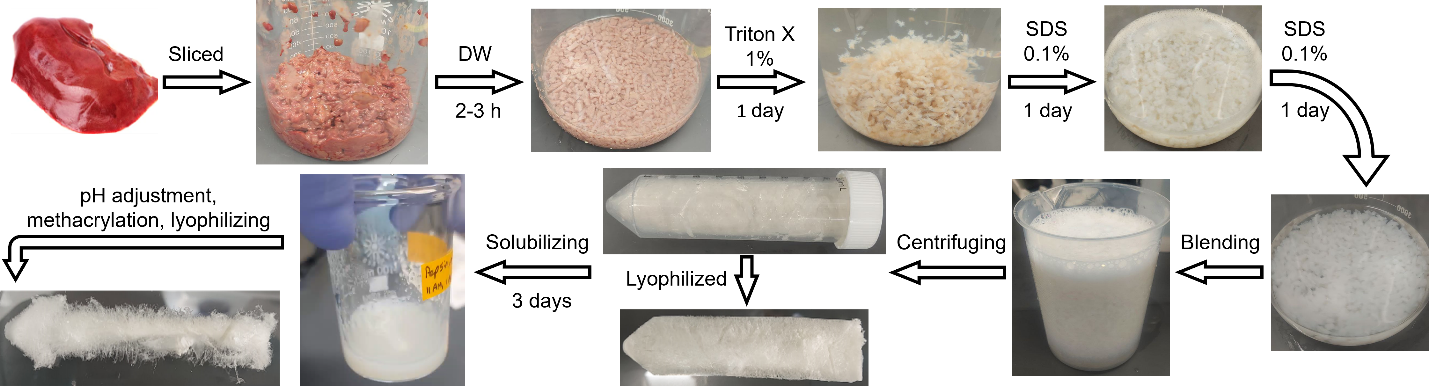


**Figure S1** LdMA synthesis process


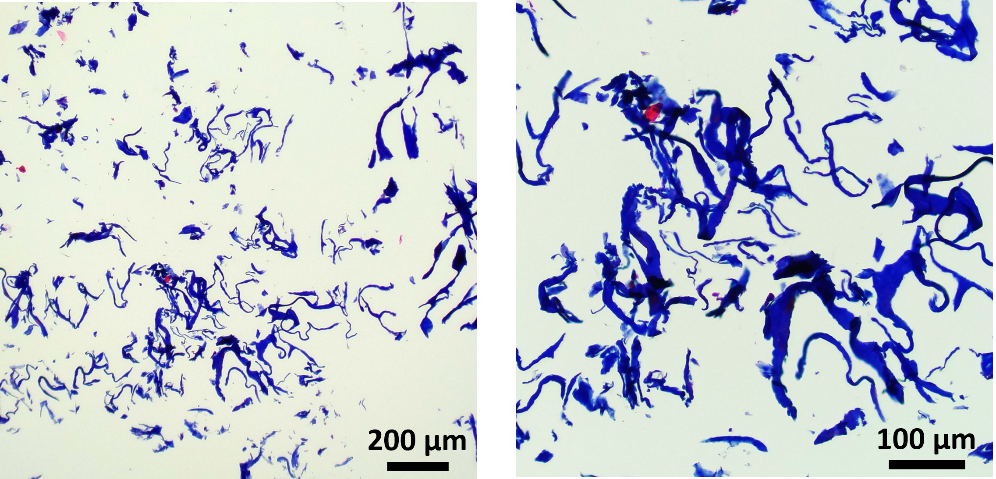


**Figure S2** LdMA Masson staining for collagen fibers


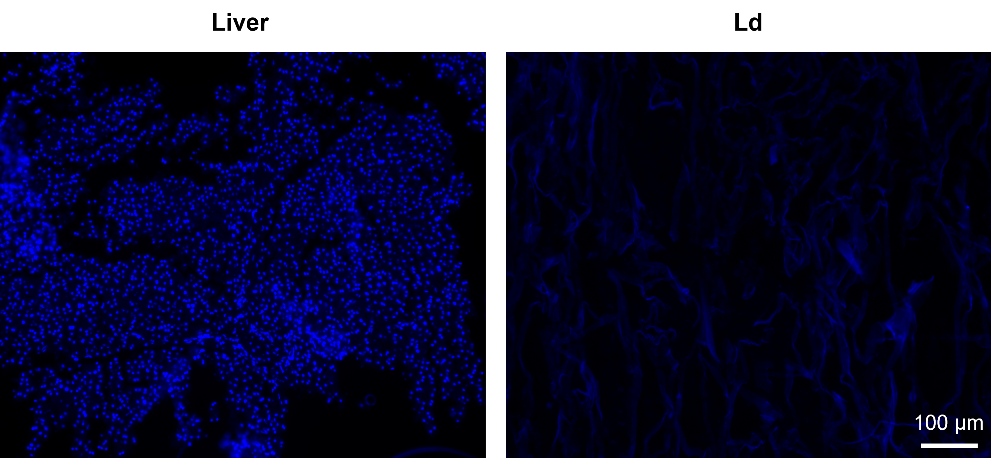


**Figure S3** Native liver and Ld DAPI staining


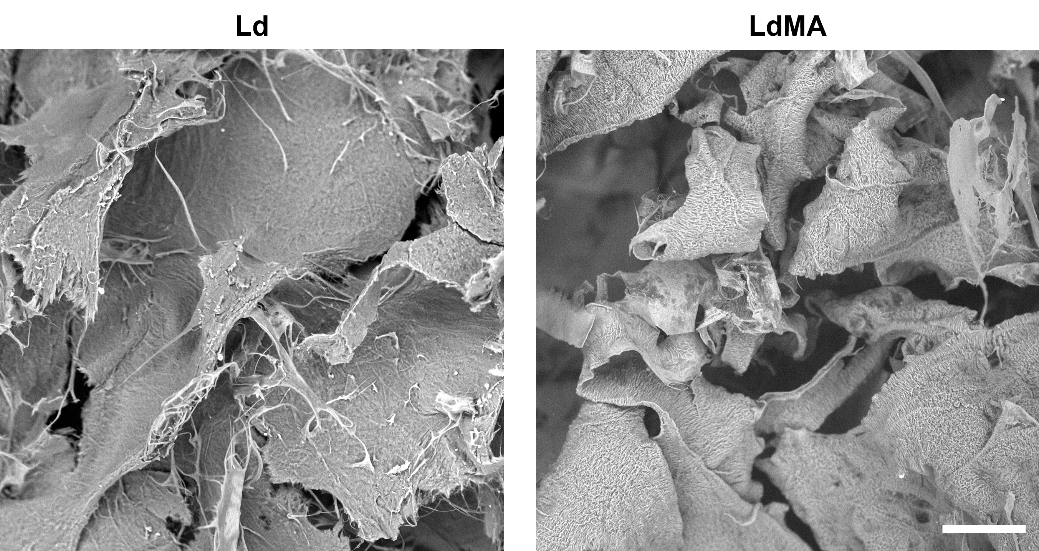


**Figure S4** Ld and LdMA SEM imaging (scale: 100 µm)


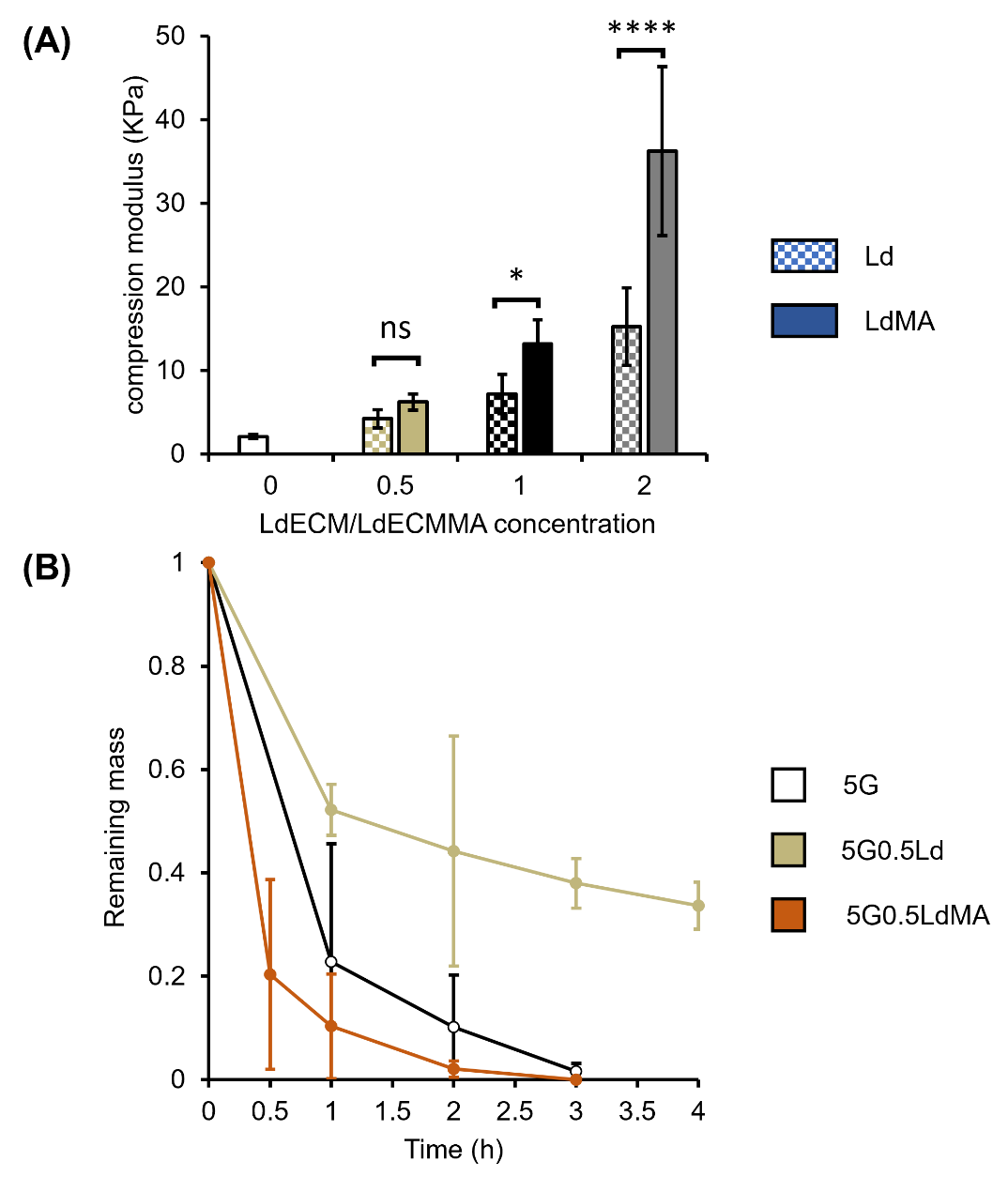


**Figure S5** dECM containing hydrogels properties evaluation, (A) GelMA-Ld hydrogels mechanical properties, and (B) degradation behavior comparison of Ld and LdMA based hydrogel


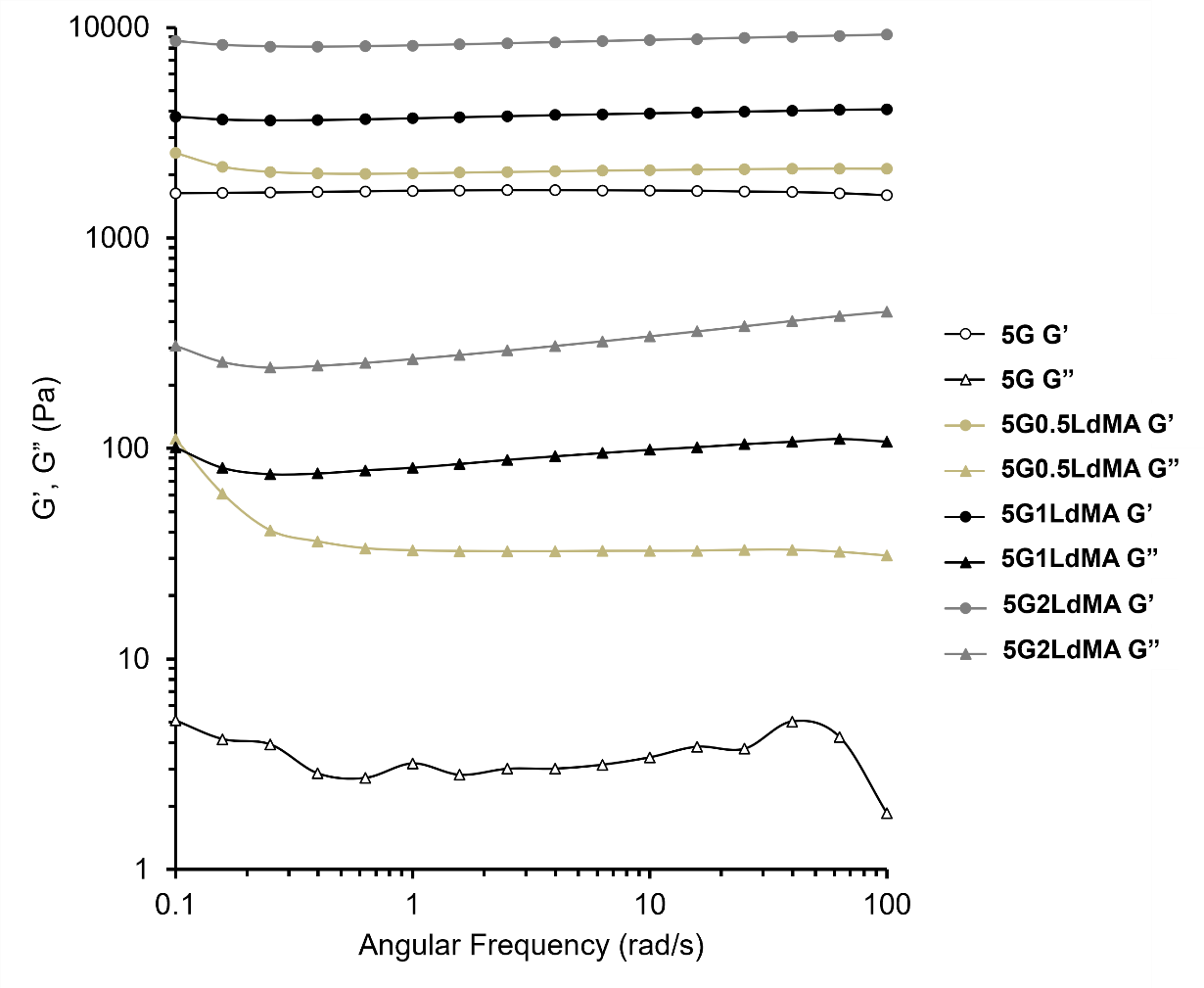


**Figure S6** Angular frequency swap evaluation of the different compositions


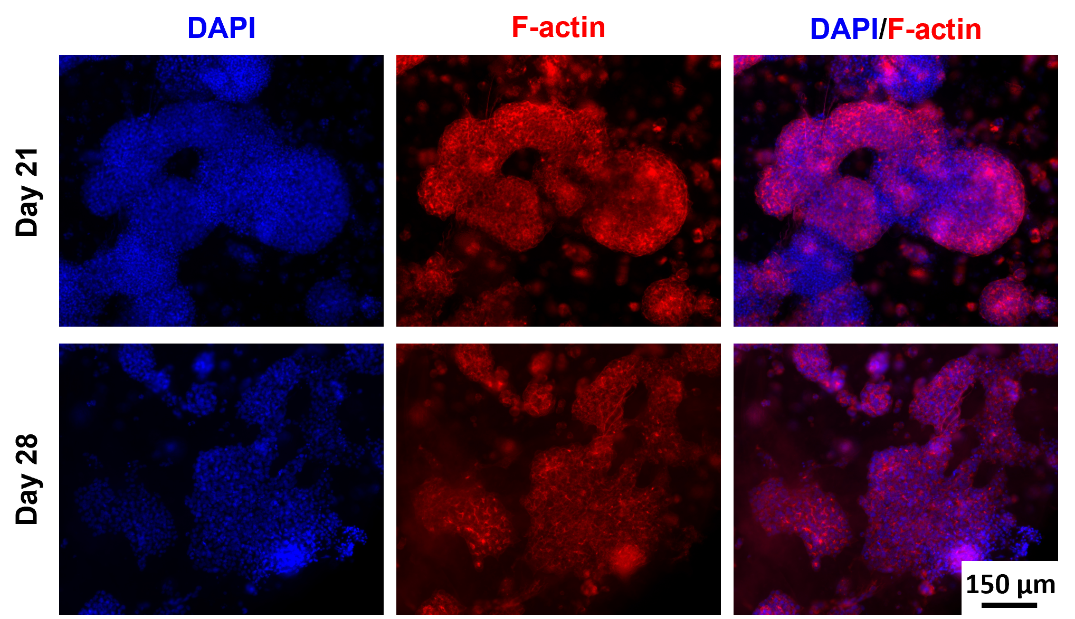


**Figure S7** Morphology evaluation of the HepG2 cells encapsulated in 5G0.5LdMA hydrogel after 21 and 28 days of the culture showing cell clusters merging


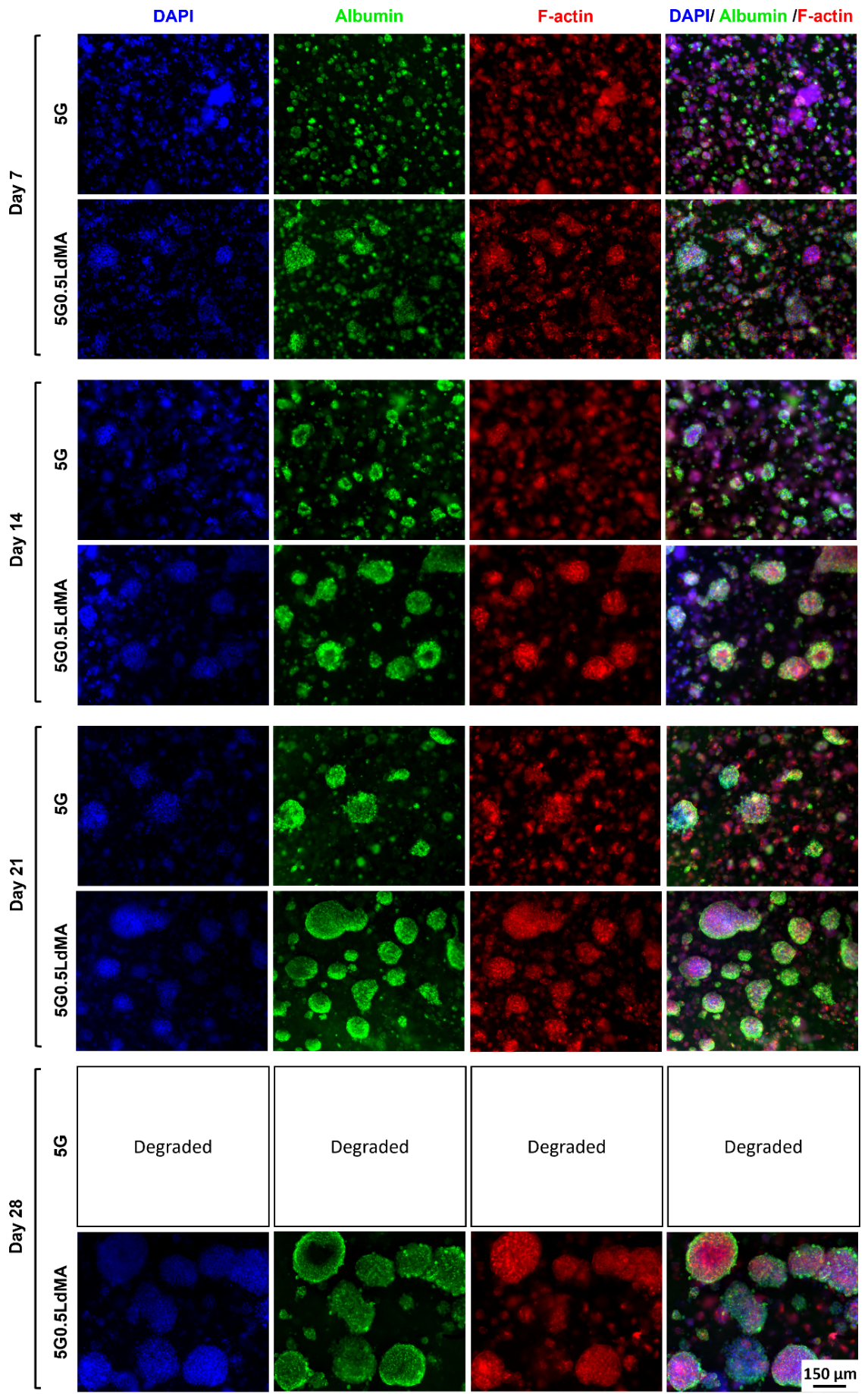


**Figure S8** Immunohistochemistary and morphological evaluation of the 3D cultured samples


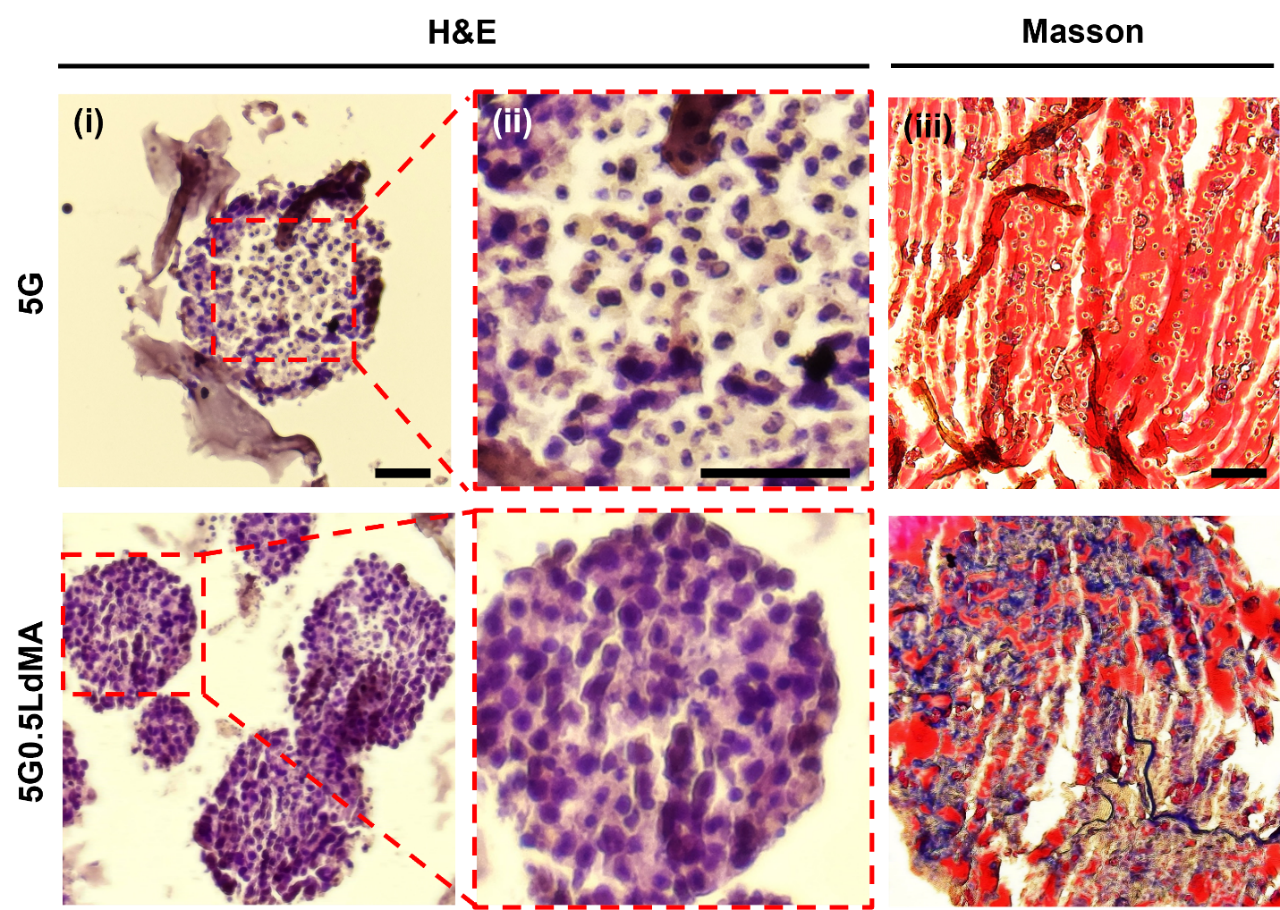


**Figure S9** Histological evaluation of biofabricated structures. H&E and Masson Trichrome staining of control and LdMA containing samples after 28 days of HepG2 cells culture (scale 50 µm)


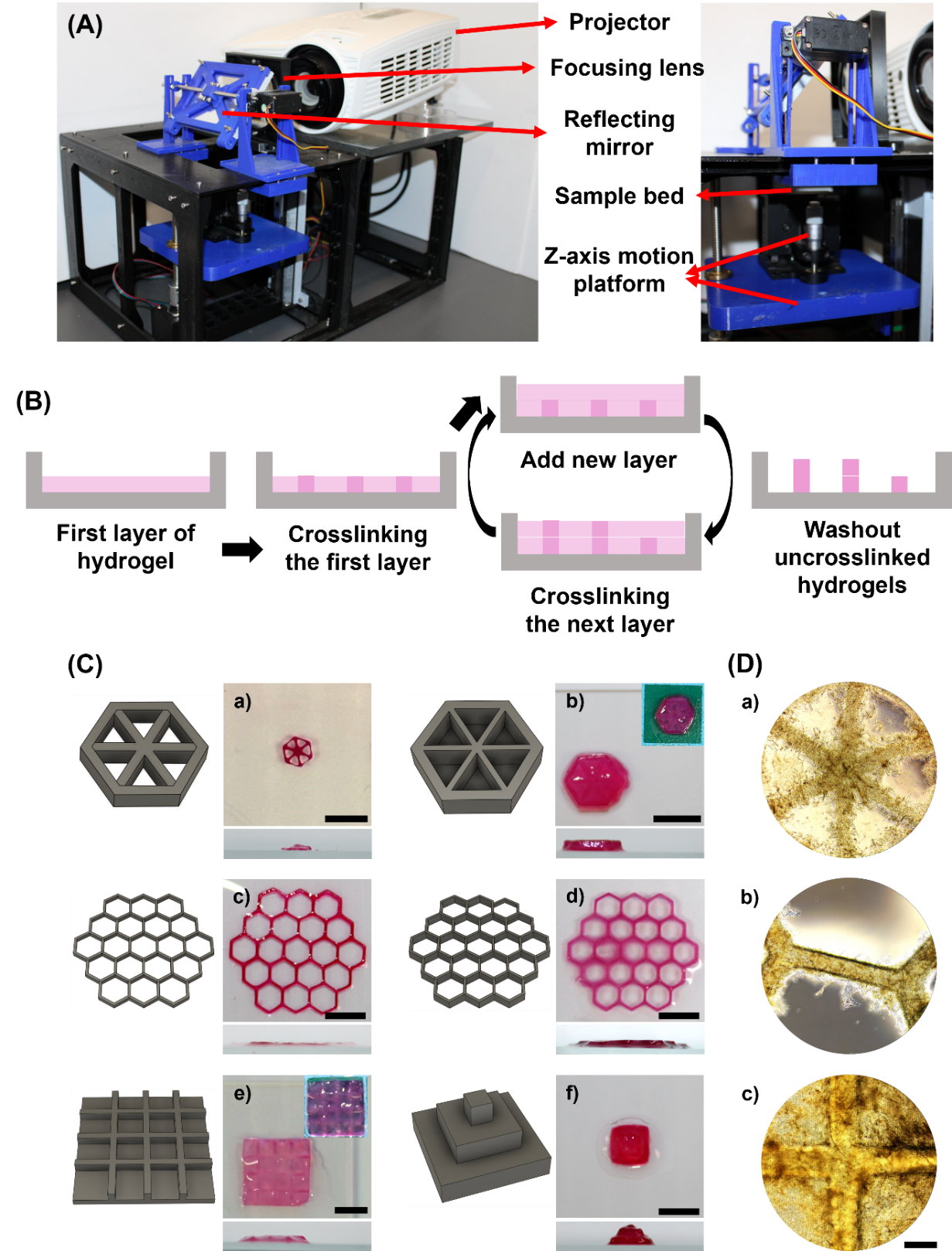


**Figure S10** 3D (bio)printing of 5G0.5dMA hydrogel, A) DLP-based bioprinting setup used for layer-by-layer additive biofabrication of 3D structures, showing the different components of the system. B) Schematic representation of the DLP (bio)printing process. C) 3D printing of various shapes with different thicknesses, showing the design, stained printed hydrogels, and side views indicating structure thickness: a) t = 0.7 mm, b) t = 1.5 mm, c) t = 0.5 mm, d) t = 1 mm, e) t = 1.2 mm, and f) t = 3 mm (scale bar = 5 mm). D) Brightfield imaging of the printed structures: a, c, and e. Scale bar = 500 µm.

**Table S1** Data obtained from rheological assessment

| sample | Gel  time (s) | Gel  point (s) | ΔG' (Pa) | Printable window (min) | ΔM (Pa) |
| --- | --- | --- | --- | --- | --- |
| 5G | ≈ 24 | ≈ 30 | 1593.40 | 4.3-4.6 | 1589.775 |
| 5G0.5LdMA | 0 | - | 2067.89779 | 4-4.2 | 2036.051 |
| 5G1LdMA | 0 | - | 3864.1969 | 3.8-4.1 | 3774.51 |
| 5G2LdMA | 0 | - | 8496.082 | 3.6-3.9 | 8224.76 |

**Table S2** Significance level of different bioinks photo patterning line thickness

| **Tukey's multiple comparisons test** | **Summary** | **Adjusted P Value** |
| --- | --- | --- |
| 5G (4 min) vs. 5G (5 min) | **** | <0.0001 |
| 5G (4 min) vs. 5G0.5LdMA (1 min) | ns | 0.1721 |
| 5G (4 min) vs. 5G0.5LdMA (2 min) | **** | <0.0001 |
| 5G (4 min) vs. 5G0.5LdMA (3 min) | **** | <0.0001 |
| 5G (4 min) vs. 5G1LdMA (30 sec) | ns | 0.0795 |
| 5G (4 min) vs. 5G1LdMA (1 min) | **** | <0.0001 |
| 5G (4 min) vs. 5G1LdMA (2 min) | **** | <0.0001 |
| 5G (4 min) vs. 5G2LdMA (30 sec) | **** | <0.0001 |
| 5G (4 min) vs. 5G2LdMA (1 min) | **** | <0.0001 |
| 5G (5 min) vs. 5G0.5LdMA (1 min) | **** | <0.0001 |
| 5G (5 min) vs. 5G0.5LdMA (2 min) | ns | 0.4014 |
| 5G (5 min) vs. 5G0.5LdMA (3 min) | **** | <0.0001 |
| 5G (5 min) vs. 5G1LdMA (30 sec) | **** | <0.0001 |
| 5G (5 min) vs. 5G1LdMA (1 min) | ns | 0.0795 |
| 5G (5 min) vs. 5G1LdMA (2 min) | **** | <0.0001 |
| 5G (5 min) vs. 5G2LdMA (30 sec) | ns | 0.9095 |
| 5G (5 min) vs. 5G2LdMA (1 min) | **** | <0.0001 |
| 5G0.5LdMA (1 min) vs. 5G0.5LdMA (2 min) | ns | 0.0666 |
| 5G0.5LdMA (1 min) vs. 5G0.5LdMA (3 min) | **** | <0.0001 |
| 5G0.5LdMA (1 min) vs. 5G1LdMA (30 sec) | ns | >0.9999 |
| 5G0.5LdMA (1 min) vs. 5G1LdMA (1 min) | ns | 0.3603 |
| 5G0.5LdMA (1 min) vs. 5G1LdMA (2 min) | **** | <0.0001 |
| 5G0.5LdMA (1 min) vs. 5G2LdMA (30 sec) | **** | <0.0001 |
| 5G0.5LdMA (1 min) vs. 5G2LdMA (1 min) | **** | <0.0001 |
| 5G0.5LdMA (2 min) vs. 5G0.5LdMA (3 min) | **** | <0.0001 |
| 5G0.5LdMA (2 min) vs. 5G1LdMA (30 sec) | ns | 0.1482 |
| 5G0.5LdMA (2 min) vs. 5G1LdMA (1 min) | ns | 0.9992 |
| 5G0.5LdMA (2 min) vs. 5G1LdMA (2 min) | **** | <0.0001 |
| 5G0.5LdMA (2 min) vs. 5G2LdMA (30 sec) | ** | 0.0087 |
| 5G0.5LdMA (2 min) vs. 5G2LdMA (1 min) | **** | <0.0001 |
| 5G0.5LdMA (3 min) vs. 5G1LdMA (30 sec) | **** | <0.0001 |
| 5G0.5LdMA (3 min) vs. 5G1LdMA (1 min) | **** | <0.0001 |
| 5G0.5LdMA (3 min) vs. 5G1LdMA (2 min) | ns | 0.3791 |
| 5G0.5LdMA (3 min) vs. 5G2LdMA (30 sec) | **** | <0.0001 |
| 5G0.5LdMA (3 min) vs. 5G2LdMA (1 min) | **** | <0.0001 |
| 5G1LdMA (30 sec) vs. 5G1LdMA (1 min) | ns | 0.5708 |
| 5G1LdMA (30 sec) vs. 5G1LdMA (2 min) | **** | <0.0001 |
| 5G1LdMA (30 sec) vs. 5G2LdMA (30 sec) | **** | <0.0001 |
| 5G1LdMA (30 sec) vs. 5G2LdMA (1 min) | **** | <0.0001 |
| 5G1LdMA (1 min) vs. 5G1LdMA (2 min) | **** | <0.0001 |
| 5G1LdMA (1 min) vs. 5G2LdMA (30 sec) | *** | 0.0005 |
| 5G1LdMA (1 min) vs. 5G2LdMA (1 min) | **** | <0.0001 |
| 5G1LdMA (2 min) vs. 5G2LdMA (30 sec) | **** | <0.0001 |
| 5G1LdMA (2 min) vs. 5G2LdMA (1 min) | ns | 0.2561 |
| 5G2LdMA (30 sec) vs. 5G2LdMA (1 min) | **** | <0.0001 |

**Table S3** IC50 values for 5G and 5G0.5LdMA after 24 and 48 h treatment with APAP

| IC50 (mM) | 2D model | 5G | 5G0.5LdMA |
| --- | --- | --- | --- |
| 24 h treatment | 12.55 | 19.20 | 44.91 |
| 48 h treatment | 1.883 | 6.132 | 9.63 |
